# Mining legume germplasm for genetic gains: An Indian perspective

**DOI:** 10.3389/fgene.2023.996828

**Published:** 2023-01-23

**Authors:** Gayacharan, Swarup K. Parida, Nupur Mondal, Rashmi Yadav, Harinder Vishwakarma, Jai C. Rana

**Affiliations:** ^1^ ICAR-National Bureau of Plant Genetic Resources, New Delhi, India; ^2^ DBT-National Institute of Plant Genome Research, New Delhi, India; ^3^ Shivaji College, University of Delhi, New Delhi, India; ^4^ Alliance of Bioversity International and CIAT, India Office, National Agricultural Science Complex, New Delhi, India

**Keywords:** pulse production, crop domestication, biotic and abiotic stresses, legume genomics, legume collections

## Abstract

Legumes play a significant role in food and nutritional security and contribute to environmental sustainability. Although legumes are highly beneficial crops, it has not yet been possible to enhance their yield and production to a satisfactory level. Amid a rising population and low yield levels, *per capita* average legume consumption in India has fallen by 71% over the last 50 years, and this has led to protein-related malnutrition in a large segment of the Indian population, especially women and children. Several factors have hindered attempts to achieve yield enhancement in grain legumes, including biotic and abiotic pressures, a lack of good ideotypes, less amenability to mechanization, poorer responsiveness to fertilizer input, and a poor genetic base. Therefore, there is a need to mine the approximately 0.4 million *ex situ* collections of legumes that are being conserved in gene banks globally for identification of ideal donors for various traits. The Indian National Gene Bank conserves over 63,000 accessions of legumes belonging to 61 species. Recent initiatives have been undertaken in consortia mode with the aim of unlocking the genetic potential of *ex situ* collections and conducting large-scale germplasm characterization and evaluation analyses. We assume that large-scale phenotyping integrated with omics-based science will aid the identification of target traits and their use to enhance genetic gains. Additionally, in cases where the genetic base of major legumes is narrow, wild relatives have been evaluated, and these are being exploited through pre-breeding. Thus far, >200 accessions of various legumes have been registered as unique donors for various traits of interest.

## 1 Introduction

**FIGURE 1:**
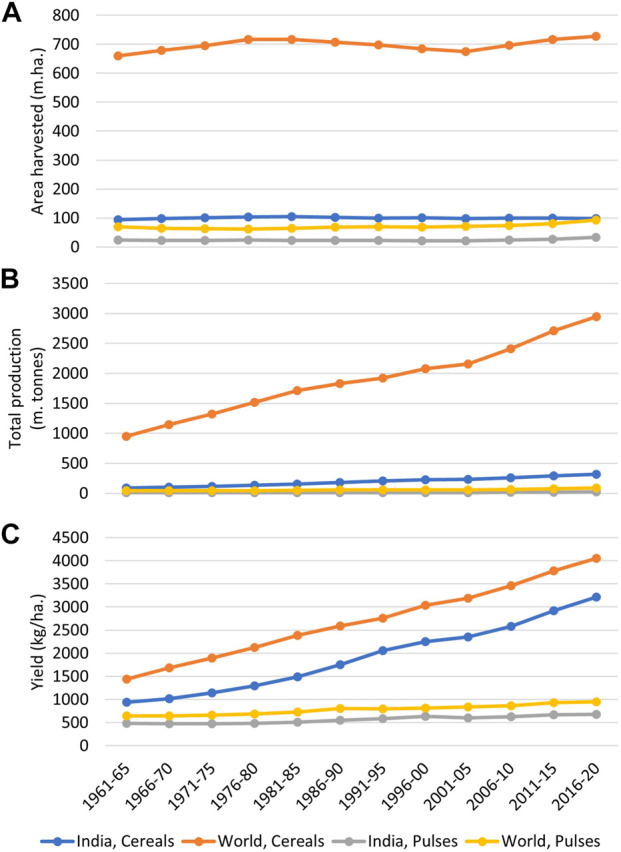
A graphical comparison of cereals and pulses in terms of total area harvested **(A)**, total production **(B)**, and yield **(C)** in India and the world. The graph indicates how the onset of the green revolution has tremendously enhanced the production of cereals in India and worldwide, which can be primarily attributed to yield improvement in these crops. By comparison, yield and production improvements in pulses have remained insignificant during this period (Data source: FAOSTAT, 2022).

Legumes of the family Fabaceae are among the most important plant groups on planet Earth. While legumes are an important source of food and nutrition, they also play an important role in improving soil health and ecosystem sustainability. Legume grains are often considered to be “the poor man’s meat,” as the vegetarian human population is highly dependent on legume grains for its protein needs (Roy et al., 2017). The “green revolution” has helped several countries to attain self-sufficiency in food, which can primarily be attributed to a manyfold increase in the production of cereals, particularly rice, wheat, and maize. However, similar advances in grain legume production have not been achieved (Figure 1), probably because legumes are less amenable to the adoption of green revolution technologies. Over 200 species of legumes are cultivated worldwide. Of these, we list the major grain legume crops, with their production and yield status and taxonomic information, in Table 1.

**TABLE 1:** Information on the production and yield status of the major grain legume crops cultivated worldwide, along with their botanical names and chromosome numbers.

Crop	Botanical name	Chromosome number n) and ploidy level x)	Production: major producing countries (million tonnes)			Countries with highest yield (kg/ha)			Total world production (MT)
			1st	2nd	3rd	1st	2nd	3rd	
Chickpea	*Cicer arietinum*	2n = 2x = 16	India (7.06)	Australia (1.32)	Myanmar (0.56)	China (5177)	Israel (4148)	Sudan (4048)	14.6
Green gram*	*Vigna radiata*	2n = 2x = 22	India (2.45)	Myanmar (1.45)	Bangladesh (0.18)	Myanmar (1,239)	Bangladesh (1,030)	Pakistan (730)	ca. 6.0
Black gram*	*Vigna mungo*	2n = 2x = 22	India (3.06)	Myanmar (1.35)	-	Myanmar (1,432)	India (546)	-	ca. 5.0
Lentil	*Lens culinaris*	2n = 2x = 14	Canada (2.64)	India (1.24)	Australia (0.52)	Jordan (3480)	China (2476)	New Zealand (2452)	6.54
Pigeon pea	*Cajanus cajan*	2n = 2x = 22	India (3.78)	Myanmar (0.44)	Malawi (0.42)	Puerto Rico (1858)	Philippines (1821)	Thailand (1701)	5.05
Field pea	*Pisum sativum*	2n = 2x = 14	Canada (4.27)	Russia (2.58)	China (1.46)	Burundi (4809)	Lebanon (4547)	Denmark (3872)	14.65
Cowpea, dry	*Vigna unguiculata*	2n = 2x = 22	Nigeria (3.66)	Niger (2.27)	Burkina Faso (0.62)	Iraq (4083)	North Macedonia (3766)	Egypt (3637)	8.35
Beans, dry	*Phaseolus* and *Vigna* spp.	-	India (5.84)	Myanmar (2.96)	Brazil (2.90)	Mali (10042)	Montenegro (6701)	Tajikistan (6451)	27.46
Broad bean	*Vicia faba*	2n = 2x = 12	China (1.74)	Ethiopia (0.98)	United Kingdom (0.58)	Argentina (8917)	Guyana (8512)	Uzbekistan (5525)	5.47
Total pulses	—	—	—	—	—	964.04	—	—	92.29

Source: FAOSTAT (https://www.fao.org/faostat/en/), as updated on 19 December 2022. Figures represent average yield and production for the period of 2016–2020.

^a^ Production and yield ^a^ata for green gram and black gram are taken from two other studies (Schreinemachers et al., 2019; Khine et al., 2021).

India is the largest producer and consumer of grain legumes globally. India’s contribution constitutes around 28.12% of global grain legume production (ca. 23.37 million tonnes), and this is the output of ca. 29 million ha of cultivated land (Department of Economics and Statistics, Department of Agriculture and Farmers Welfare, Ministry of Agriculture and Farmers Welfare, GoI). Of this total, around 34% of the cultivated land (9.89 mha) is covered by cultivation of chickpea alone; this is followed by black gram (4.81 mha), pigeon pea (4.72 mha), green gram (4.61 mha), lentil (1.43 mha), and field pea (0.75 mha). Other minor legumes cultivated in India are green gram, cowpea, moth bean, grass pea, and horse gram. In 2020, global grain legume production was approximately 83.1 million tonnes; this figure is the sum of the production values of grain legumes grown globally, specifically beans (dry), chickpeas, peas (dry), lentils, cowpeas, pigeon peas, Bambara beans, and other minor pulses (www.fao.org/faostat/en). The primary contributors to this total (at 42.12 million tonnes) were the five major grain legume producing countries of India (28.12%), Canada (9.24%), Myanmar (4.84%), Nigeria (4.47%), and the Russian Federation (4.01%). The major producing countries for each crop are given in Table 1.

Globally, over 3,800 improved cultivars of grain legumes have been released, with improvements to traits such as yield, crop duration, and nutritional qualities (Pratap et al., 2022). However, between 1961 and 2020, only a 1.5-fold increase in grain legume productivity was achieved, from 637 kg/ha to 964 kg/ha (Pratap et al., 2022; Figure 1). This can be primarily attributed to various factors, such as a narrow genetic base in cultivated gene pools, poor plant ideotype, high susceptibility to insect pests and diseases, a lack of robust seed systems, and frequent stresses from drought, heat, and flooding.

Over 850 high-yielding varieties of food legumes have been developed in India, and these are now playing a vital role in food legume production (Chauhan et al., 2016). However, the foundation of any crop breeding program is based on only a small number of parental lines, which has led to a narrow genetic base in these cultivated varieties. In a pedigree analysis, it was found that 41% of chickpea varieties had PB 7 as one of its ancestors; in pigeon pea, T 1 and T 190 appeared in 34% of varieties; and T 9 and T 1 appeared in 64% and 35% of varieties of black gram and green gram, respectively (Kumar et al., 2004). Furthermore, in the process of rigorous selection in the development of a variety, alleles conferring defense mechanisms are also lost. This is one of the reasons that the actual yield of most food legume crops is half their potential yield. Recently, drastic climatic change, to which abrupt temperature rises, erratic and heavy rainfall, frequent droughts, episodes of flooding, and rapid pest and pathogen evolution can be attributed, has exceeded the adaptation capability of modern varieties (Guo, 2022). As a result, the breakdown of resistance to biotic stress has become rather common in modern cultivars (Sharma et al., 1999; Burdon et al., 2014; Rex Consortium, 2016; Mbinda and Masaki, 2021; Hu et al., 2022; Van de Wouw et al., 2022). Therefore, *ex situ* collections are now being utilized to increase genetic variability in modern cultivars in order to improve their climate resilience, including *via* genetic gains in breeding programs. Advances in genomics, phenomics, and breeding methods are playing an important role and exerting a significant impact on legume improvement by accelerating genetic gains *via* enhancements to selection efficiency and the advancement of desired genotypes with high precision.

**FIGURE 2:**
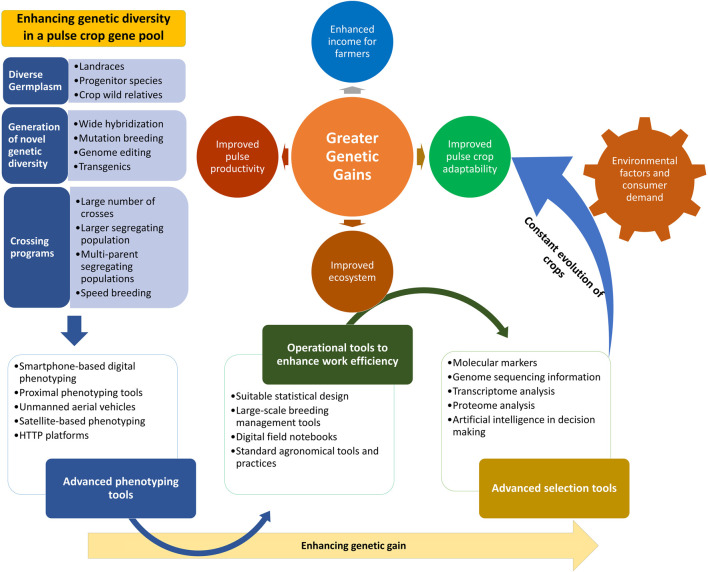
Strategy to enhance genetic gains through utilization of advanced phenotyping tools, efficient operational tools, and advanced selection methods and technologies. A strategy to achieve higher genetic gains by broadening the genetic base through the infusion of increasing levels of variability from diverse sources into the target breeding populations is illustrated. The integration of improved crossing program strategies and advanced tools for phenotyping, operations, and desired genotype selection will further enhance the genetic gains made. This strategy will help with the attainment of greater genetic gains along with enhanced crop adaptability to changing climatic conditions.

It is well understood that, in terms of enhancing the variability of a crop gene pool, landraces are the primary resource; the desired traits need to be sought out among these, as they are easy to cross and their use significantly reduces the chances of linkage drag as compared to the use of wild species. Additionally, landraces are well adapted to microclimatic niches and have several superior traits in terms of nutritional value. In this study, we have focused on the identification of desired genes and traits and their utilization in the improvement of legume crops. We also propose a comprehensive strategy for the enhancement of genetic gains (Figure 2).

## 2 Legume germplasm collections in the Indian national gene bank

The collection, conservation, and selection of germplasm are the primary components of the crop domestication process. Wild species were initially brought under cultivation and improved through selection for their agronomic traits, and the practice is still being followed by farmers and breeders. Diverse environments of crop cultivation, including rainfed, dryland, and coastal areas, flood-prone areas, and areas at high altitude, as well as disease hotspots and human preferences in terms of nutritional qualities, aesthetics, and cultural values, have played important roles in the development and deployment of diverse germplasm. Although diversity has been continually developing and has been sustained through traditional practices over the last several thousand years, crop diversity has recently come under threat due to increasing pressure arising from demographic, sociocultural, and technological changes.

India is rich center of diversity for several cultivated crops, including important legumes such as chickpea (*Cicer arietinum*), moth bean (*Vigna aconitifolia*), rice bean (*Vigna umbellata*), cowpea (*Vigna unguiculata*), yard-long bean (*Vigna unguiculata* subsp. *sesquipedalis*), green gram (*V. radiata*), black gram (*V. mungo*), horse gram (*Macrotyloma uniflorum*), and dolichos bean (*Lablab purpureus*) (Zeven and Zhukovsky, 1975; Hawkes, 1983). The development of extensive and organized germplasm collections and conservation activity in India began only after the establishment of the National Bureau of Plant Genetic Resources (NBPGR) in 1976 (Rana et al., 2016). Since then, around 63,000 accessions of legumes have been collected and conserved in *ex situ* conditions (Table 2). The organization is continuously enriching its collections based on gap analysis with respect to earlier collections established within India and also introducing accessions from abroad. Globally, over 0.7 million legume germplasms, including their crop wild relatives (CWRs), are conserved in 276 gene banks distributed worldwide (WIEWS, 2022).

**TABLE 2:** Status of collections of grain legume crops and their wild relatives available in the Indian National Gene Bank.

Crop/species name	Exotic	Indigenous	Total
**Chickpea (*Cicer arietinum*)**	2961	11452	14413
***Cicer* wild species** *C. bijugum* (31), *C. chorassanicum* (2), *C. cuneatum* (6), *C. echinospermum* (18), *C. judaicum* (54), *C. microphyllum* (35), *C. pinnatifidum* (27), *C. reticulatum* (18), *C. yamashitae* (4), unknown species (9)	148	56	204
**Pigeon pea (*Cajanus cajan*)**	306	10904	11210
***Cajanus* wild species** *C. cajanifolius* (2), *C. albicans* (3), *C. scarabaeoides* (49), *C. volubilis* (1), *C. sp.* (2), *Atylosia* (18), *Rhynchosia aurea* (1), *R. bracteata* (1), *R. himalensis* (1), *R. minima* (10), *R. sublobata* (4)	0	92	92
**Lentil (*Lens culinaris*)**	556	1835	2391
**Other *Lens* species** *L. culinaris* subsp*. odemensis* (29), *L. culinaris* subsp*. orientalis* (63), *L. culinaris* subsp*. tomentosus* (6), *L. esculenta (15), L. lamottei* (3), *L. ervoides* (67), *L. nigricans* (21), *L. odemensis* (6)	202	8	210
**Pea (*Pisum sativum*)**	1,082	3075	4157
**Other *Pisum* species** *Pisum sativum* subsp*. hortense* 7), *Pisum sativum* var*. arvense* (260)	25	242	267
**Green gram (*Vigna radiata*)**	535	3406	3941
**Black gram (*Vigna mungo*)**	5	2096	2097
**Cowpea (*Vigna unguiculata*)**	1063	2583	3646
**Moth bean (*Vigna aconitifolia*)**	37	1472	1509
**Rice bean (*Vigna umbellata*)**	144	1883	2027
**Adzuki bean (*Vigna angularis*)**	97	89	186
**Yard-long bean (*Vigna unguiculata* subsp. *sesquipedalis*)**	1	128	129
***Vigna* wild species** *Vigna radiata* var*. sublobata* (228), *V. radiata* var*. setulosa* (3), *V. mungo* var*. silvestris* (17), *V. angularis* var*. nipponensis* (9), *V. bourneae* (4), *V. dalzelliana* (30), *V. hainiana* (6), *V. khandalensis* (1), *V. membranacea* (1), *V. minima* (1), *V. nepalensis* (3), *V. parkeri* (2), *V. pilosa* (4), *V. racemosa* (2), *V. reticulata* (1), *V. stipulacea* (6)*, V. trilobata* (144), *V. trinervia* (2), *V. trinervia* var*. bourneae* (11), *V. vexillata* (109), *V. marina* (2), *V. wightii* (1), *Vigna sp.* (13)	9	591	600
**Common bean (*Phaseolus vulgaris*)**	1,669	2236	3905
**Horse gram (*Macrotyloma uniflorum*)**	11	3122	3133
**Grass pea (*Lathyrus sativus*)**	90	2524	2614
**Fava bean (*Vicia faba*)**	354	500	854
**Total**	**9295**	**48294**	**57585**

Source: Indian National Gene Bank database (http://www.nbpgr.ernet.in:8080/PGRPortal).

Bold values in the first column are legume crops followed by their related wild species.

## 3 Utilization of grain legume germplasm for crop improvement

Crop evolution in early times was based entirely on appearance and performance in terms of agro-morphological traits, and these are still the primary focus of plant breeders and researchers. During the domestication process and subsequent structured breeding programs, genotypes with greater biotic and abiotic stress tolerance are often unintentionally selected, but agronomic traits have always been the prime target for selection. Landraces, which are locally adapted cultivars with a high level of genetic variability developed by farmers over the years, are the primary source of such traits in modern breeding programs. However, in terms of the utilization of germplasm from gene banks, it has become difficult to identify a manageable number of accessions with the desired levels of variability and traits. Recognizing this challenge, Frankel (1984) proposed the concept of a core collection, a minimum number of representative accessions representing maximum variability across the entire collection. Since then, several crop-specific diverse core sets have been developed (Table 3), and this has accelerated the utilization of gene bank collections. A number of significant studies conducted to date in the area of trait identification and utilization are discussed below, presented in crop-wise fashion, and promising trait-specific accessions are summarized in Table 4 (biotic stress resistance) and Table 5 (abiotic stress tolerance). We also find that in the process of breeding modern varieties, the focus on yield *per se* has eventually led to a gradual decrease in the nutritional qualities of new varieties. Comparative studies on the nutritional composition of landraces and traditional cultivars in various crops, such as vegetables and fruits (Davis et al., 2004), wheat (Fan et al., 2008), the potato (White et al., 2009), the common bean (Celmeli et al., 2018), and green gram (Ebert et al., 2017), have indicated that the improved varieties are poorer than the older varieties in terms of nutritional value. Therefore, recognizing the significance of nutritional value and of the availability of nutritional variability in germplasm, we also discuss the important nutritional characteristics of each legume crop in the following sections.

**TABLE 3:** List of core collections developed for grain legume crops.

Crop	Core/mini-core collection size	Base accessions	Traits	References
Chickpea	1956	16,991	13 morphological quantitative traits; passport information	Upadhyaya et al. (2001)
	211^a^	1,956	22 morphological and agronomic traits	Upadhyaya and Ortiz, (2001)
	1,103	14,651	Eight quantitative and 12 qualitative agro-morphological traits	Archak et al. (2016)
Pigeon pea	1,290	12,153	Geographic origin; 14 qualitative morphological traits	Reddy et al. (2005)
	146^a^	1,290	18 qualitative and16 quantitative traits	Upadhyaya et al. (2006)
Lentil	287	2,390	Documented diversity	Simon & Hannan, (1995)
	170	2,324	26 agro-morphological traits	Tripathi et al. (2021a)
Green gram	1,481	5,234	Geographic origin; 8 quantitative traits	Schafleitner et al. (2015)
	152	1,532	Geographical origin; 19 quantitative and 19 qualitative traits	Bisht et al. (1998)
	289*	1,481	Phenotypic and SSR genotypic data	Schafleitner et al. (2015)
Adzuki bean	96	616	13 SSR molecular markers	Xu et al. (2008)
Common bean	171	423	Seed coat traits; geographical information; 46 SSR markers	McClean et al. (2012)
	300	544	Geographical information; morphological traits; phaseolin seed protein	Logozzo et al. (2007)
	52	388	Agro-morphological traits; phaseolin seed protein	Rodiño et al. (2003)
Cowpea	2062	12,000	Geographical information; 28 agro-botanical traits	Mahalakshmi et al. (2007)
Pea	48	731	21 SSR markers	Xu-Xiao et al. (2008)
Sem	46	249	28 agro-morphological traits	Pengelly & Maass, (2001)

^a^ Mini-core.

**TABLE 4:** List of important resistance sources identified for various important biotic stresses in grain legume crops.

Crop	Trait	Screened germplasm	Screening method	Sources identified	References
Chickpea	*Fusarium* wilt resistance	13,500	Field and pot conditions	160 accessions	Haware et al. (1992)
		414 germplasm/varieties	Field conditions in sick plot	35 accessions	Chaudhry et al. (2007)
		1,915 accessions of Kabuli type	Field sick plot and laboratory conditions	110 accessions	Halila and Strange, (1997)
		5,174	Screened at ICARDA	110 accessions	Singh, (1997)
	Ascochyta blight resistance	1,970 diverse germplasm	Field conditions in sick plot, multiple seasons	IC275447, IC117744, EC267301, IC248147, and EC220109	Gayacharan et al., 2020c
		19,375 germplasm	Screened at ICARDA	32 accessions	Singh, (1997)
	Collar rot resistance	98	Greenhouse conditions	FLIP 97–132C, FLIP 97-85C, FLIP 98-53C, ILC -5263, and NCS 9905	Akram et al. (2008)
Green gram	MYMV resistance	100 germplasm lines	Field conditions	014043, 014133, 014249, 014250	Iqbal et al. (2011)
		81 germplasm lines	Field conditions	IC76361, IC119020-1, PLM490, IC75200, IC119020-2, CO7, CO8	Nainu and Murugan, (2020)
		120 germplasm lines	Field conditions	EC 398897, TM-11-07, TM-11-34, PDM-139, and 6 others	Mohan et al. (2014)
	Bruchid beetle tolerance	335 germplasm lines	‘Free choice’ and ‘no choice’ test method	LM 131, V 1123, LM 371, and STY 2633	Duraimurugan et al. (2014)
	Spotted pod borer (*Maruca vitrata*) tolerance	110 germplasm lines	Field conditions	KM-9-128, KM-9-136, RMG-492, LGG-527, and LGG-538	Sandhya et al. (2014)
	Bean fly (*Ophiomyia phaseoli*) tolerance	3,713 germplasm lines	Field conditions	28 accessions	Chiang and Talekar, (1980)
Black gram	MYMV resistance	344 germplasm lines	Field conditions and artificial agro-inoculation	IC144901 and IC001572	Bag et al. (2014)
		128 germplasm lines	Field conditions	KU 96-3, NDU 12-1, NIRB 002, NIRB 003, and NIRB 004	Kumari et al. (2020)
	ULCV resistance	87 germplasm lines	Field conditions	2cm-703, 90cm-015, 93cm-006, 94cm-019, 99cm-001, IAM 382-1, IAM382-9, IAM382-15, and IAM133	Ashfaq et al. (2007)
	Bruchid beetle tolerance	140 germplasm lines	‘Free choice’ and ‘no choice’ test method	UH 82-5, IC 8219, and SPS 143	Duraimurugan et al. (2014)
Moth bean	MYMV resistance	180 germplasm lines	Field conditions, multiple seasons	IC36522 and IC36217	Singh et al. (2020)
		204 diverse germplasm lines	Field conditions	PLMO 12, IC 36096, IC 415152, IC 129177, IC 129177, and 9 others	Meghwal et al. (2015)
	Leaf crinkle virus	180 germplasm lines	Field conditions, multiple seasons	IC39786 and IC39822	Singh et al. (2020)
		44 germplasm lines	Field conditions, multiple seasons	IC39786	Vir and Singh, (2015)
	*Cercospora* leaf spot resistance	180 germplasm lines	Field conditions, multiple seasons	IC16218	Singh et al. (2020)
Cowpea	Aphid (*Aphis craccivora*) resistance	105 cultivated and 92 wild germplasm	Greenhouse conditions	TVNu 1158	Souleymane et al. (2013)
	Bacterial blight (*Xanthomonas axonopodis* pv. *vignicola*) resistance	50 improved cultivars	Artificial inoculation	DANILA, IT00K-1263, IT03K-324-9, and 11 others	Boukar et al. (2019)
	Cowpea Mosaic Virus (CMV) resistance	225 germplasm lines	Field conditions, multiple seasons	IC202786, IC202809, and Bellary local	Deshpand et al. (2010)
	*Cercospora* leaf spot resistance	225 germplasm lines	Field conditions, multiple seasons	IC257420, IC27502, IC91556, IC198330, IC202797, IC219574, and IC202791	Deshpand et al. (2010)
	Cowpea rust (*Uromyces vignae*) resistance	225 germplasm lines	Field conditions, multiple seasons	IC206240, IC214834, IC214835, IC219871, Guntur local, and Bellary local	Deshpand et al. (2010)
	Bruchid (*Callosobruchus maculatus*) resistance	103 germplasm lines	No-choice test method	EC528425 and EC528387	Tripathi et al. (2020)
Lentil	Wilt (*Fusarium oxysporum* f.sp. *lentis*) resistance	196 landraces	Controlled and field conditions	BGE016363, BGE019696, BGE019698, BGE019708, and 8 others	Pouralibaba et al. (2015)
		93 diverse germplasm lines	Greenhouse and sick plot conditions	IG 69549 and IG 70238	Meena et al. (2017)
	Rust (*Uromyces fabae* (Pers.) de Bary) resistance	321 germplasm lines	Glasshouse and field conditions, multiple locations	Precoz, L 1534, L 2991, L 178, L 2297, L 24123, and HPLC 8868	Kumar et al. (1997)
		286 germplasm lines	Growth chamber conditions	RR-107, ILL7207, ILL7716, and ILL7618	Rubiales et al. (2013)
	Blight (*Stemphylium botryosum* Wallr.) resistance	70 germplasm lines including wild	Growth chamber, greenhouse, and field conditions	Various promising accessions identified	Podder et al. (2013)
	Seed weevil (*Bruchus spp*.) resistance	571 germplasm lines including wild	Field conditions with artificial release of insects	32 accessions	Laserna-Ruiz et al. (2012)
	Root-knot nematode (*Meloidogyne incognita*) resistance	300 germplasm lines	Pot conditions, artificial inoculation	EC223269, EC076551-C, EC267577-D, EC267555, EC255504, and 4 others	Khan et al. (2017)
Pigeon pea	*Fusarium* wilt and sterility mosaic disease resistance	146 germplasm accessions of a mini-core collection	Artificial field epiphytotic conditions, multiple seasons	ICP 6739, ICP 8860, ICP 11015, ICP 13304, and ICP 14819 have combined resistance	Sharma et al. (2012)
	*Fusarium* wilt (*Fusarium udum*) resistance	104 germplasm lines	Greenhouse and field conditions	VBG 42, VBG 52, and VBG 57	Okiror, (1999)
	Sterility mosaic disease resistance	976 accessions	Artificial epiphytotic conditions, multiple seasons	ICPLs 20094, 20106, 20098, and 20115	Sharma et al. (2015)
		88 germplasm lines	Field conditions at 10 locations	ICP 7867, ICP 10976, and ICP 10977	Nene et al. (1989)
		60 accessions of *C. carabaeoides*	Leaf-stapling followed by petiole grafting	ICP15684, ICP15688, ICP15692, ICP15695 and others	Kulkarni et al. (2003)
	Spotted pod borer (*Maruca vitrata*) tolerance	271 germplasm lines	Open field screening nursery	Promising accessions from four determinate and 12 nondeterminate types	Saxena et al. (2002)
Field pea	Pea weevil (*Bruchus pisorum* L.) tolerance	602 germplasm lines	Field conditions at three locations	Ethiopian gene bank acc. 32454, 235002, 226037, and 32410	Teshome et al. (2015)
	Powdery mildew (*Erysiphe pisi*) resistance	701 germplasm lines	Natural epiphytotic conditions	EC598655, EC598878, EC598704, IC278261, and IC218988	Rana et al. (2013)
	Rust (*Uromyces viciae-fabae*) resistance	250 lines consisting of released varieties, germplasm accessions, and advance breeding lines	Multilocation, field conditions, and further validation of 23 selected lines	IPF-2014-16, KPMR-936, and IPF-2014-13	Das et al. (2019)
Common bean	Angular leaf spot (*Phaeoisariopsis griseola*) resistance	1944 diverse germplasm lines including wild	Field and screenhouse conditions	Various resistance sources identified	Mahuku et al. (2003)
		300 germplasm lines	Field conditions	14 resistant accessions	Rezene and Mekonin, (2019)
	Damping-off (*Rhizoctonia solani*) resistance	274 germplasm lines	Artificial inoculation, pot conditions	PI 310668 and PI 533249	Peña et al. (2013)
	*Fusarium* root rot (*Fusarium cuneirostrum*) resistance	248 wild germplasm	Greenhouse, small pots, and artificial inoculation	PI417775 highly resistant; 21 others resistant	Haus et al. (2021)
	*Fusarium* wilt (*Fusarium oxysporum*) resistance	248 wild germplasm	Greenhouse, small pots, and artificial inoculation	PI661845 and PI535441 highly resistant; 16 others resistant	Haus et al. (2021)
	Bacterial wilt (*Curtobacterium flaccumfaciens* pv*. flaccumfaciens*) resistance	467 diverse germplasm	Pot conditions, artificial inoculation	PI 325691	Urrea & Harveson, (2014)

**TABLE 5:** List of important resistance sources identified for various important abiotic stresses in grain legume crops.

Crop	Trait	Germplasm screened	Screening method	Sources identified	References
Chickpea	Salinity tolerance	600 selected based on various strategies	Pot conditions	33 lines	Maliro et al. (2008)
		211 acc. of chickpea mini-core collection and 41 popular varieties	Pot conditions with 100 mM NaCl solution to field capacity of the soil	ICC 10755, ICC 13124, ICC 13357, ICC 15406, ICC 15697, and 5 others	Serraj et al. (2004)
		180 germplasm lines	Paper cup, greenhouse conditions, 8 ds/m electrical conductivity	ICCV 00104, ICCV 06101, CSG8962, and JG62	Kumar et al. (2016)
	Heat stress tolerance	References set of 200 accessions having very long duration	Late-sown, field conditions	18 lines	Krishnamurthy et al. (2011)
		167 accessions	Late- vs. timely-sown, field conditions	ICCV 95311, ICCV 98902, ICCV 07109, ICCV 92944, ICC 6969, and 5 others	Devasirvatham et al. (2015)
		35 early-maturing lines	Late- vs. timely-sown, field conditions	ICC 14346	Upadhyaya et al. (2011a)
	Drought tolerance	211 accessions of mini-core collection	Field conditions	ICC 867, ICC 1923, ICC 9586, ICC 12947, and ICC 14778	Krishnamurthy et al. (2010)
		1,500 diverse germplasm	Field conditions	ICC4958	Saxena et al. (1993)
		150 Kabuli type germplasm	Field conditions	MCC544, MCC696, and MCC693	Ganjeali et al. (2011)
	Cold tolerance	14 accessions	Field and controlled environments	ICCV 88502 and ICCV 88503	Srinivasan et al. (1998)
		3,276 germplasm and breeding lines	Field conditions	21 lines	Singh et al. (1989)
Green gram	Heat stress tolerance	41 elite lines	Late- vs. timely-sown, field conditions	EC693357, EC693358, EC693369, Harsha, and ML1299	Sharma et al. (2016)
	Drought tolerance	100 diverse germplasm	Hydroponics in controlled conditions	IC333090 and IC507340	Meena et al. (2021)
Black gram	Salt tolerance	48 genotypes	Various salinity levels at seedling stage	VNBG 017, AUB 3, and AUB 20	Priyadharshini et al. (2019)
	Waterlogging tolerance	290 germplasm lines	Pot conditions, 10 days of flooding 30 days after sowing	IC530491 and IC519330	Bansal et al. (2019)
Moth bean	Drought tolerance	32 diverse germplasm	Withdrawal of irrigation, field conditions	IC129177, IC103016, IC415139, IC 415155, IC36157, Maru moth, and Jadia	Malambane and Bhatt, (2014)
		15 diverse germplasm	Withdrawal of irrigation, field conditions	IC103016, IC36011, and IC36157	Sachdeva et al. (2016)
Cowpea	Drought tolerance	1,288 randomly selected lines	Withdrawal of irrigation, field conditions	TVu1436, TVu9693, TVu12115, TVu14632, and TVu15055	Fatokun et al. (2012)
	Salt tolerance	151 germplasm lines	Artificial conditions with 150 mM NaCl application at germination stage	PI582422, 09–529, PI293584, and PI582570	Ravelombola et al. (2017)
		155 germplasm lines	Artificial conditions with 200 mM NaCl application at seedling stage	PI354686, PI353270, PI354666, and PI354842	Dong et al. (2019)
		116 acc. at germination stage and 155 acc. at seedling stage	Artificial conditions with 150 and 200 mM NaCl application at germination and seedling stage screening, respectively	Trait-specific promising genotypes	Ravelombola et al. (2018)
	Heat tolerance	130 germplasm lines	Field conditions, multiple seasons	EC472250, EC472267, EC$&2285, EC472286, EC472289, and Pusa Komal	Mishra et al. (2005)
Lentil	Combined terminal heat and drought stress tolerance	166 selected through FIGS^#^	Field conditions at two contrasting locations	ILL 7835, ILL 6075, ILL 6362, ILL 7814, ILL 7835, and ILL 7804	Rajendran et al. (2020)
	Boron tolerance	310 germplasm lines	Field conditions at seedling stage	ILL213A and ILL2024	Hobson et al. (2006)
	Salt tolerance	133 germplasm lines	Germination and seedling stage, NaCl application	ILL 5845, ILL 6451, ILL 6788, ILL 6793, and ILL 6796	Ashraf and Waheed, (1990)
Pigeon pea	Waterlogging tolerance	272 diverse accessions	*In vitro* laboratory conditions and natural field conditions	ICPH 2431, ICPH 2740, ICPH 2671, and 9 others	Sultana et al. (2013)
		146 accessions of mini-core collection	Pots placed in water tanks, multiple durations and seasons	24 accessions	Krishnamurthy et al. (2012)
Adzuki bean	Drought tolerance	80 germplasm lines	Mannitol-induced drought stress	-	Zhu et al. (2019)
Field pea	Cold tolerance	3,672 germplasm lines	Field conditions	214 accessions	Zhang et al. (2016)
	Frost tolerance	83 accessions collected from 34 countries	Controlled environmental chamber	ATC 104, ATC 377, ATC 968, ATC 3992, and ATC 4204	Shafiq et al. (2012)
	Salinity tolerance	780 globally distributed germplasm	Artificial conditions, using NaCl	ATC1836	Leonforte et al. (2013)
	High temperature tolerance	150 genotypes	Field conditions; timely, moderately late, and very late sowing	IPFD 11-5, Pant P-72, P-1544-1, and HUDP 11	Lamichaney et al. (2021)

#FIGS: focused identification of ermplasm strategy.

### 3.1 Chickpea

#### 3.1.1 Agronomic traits

**FIGURE 3:**
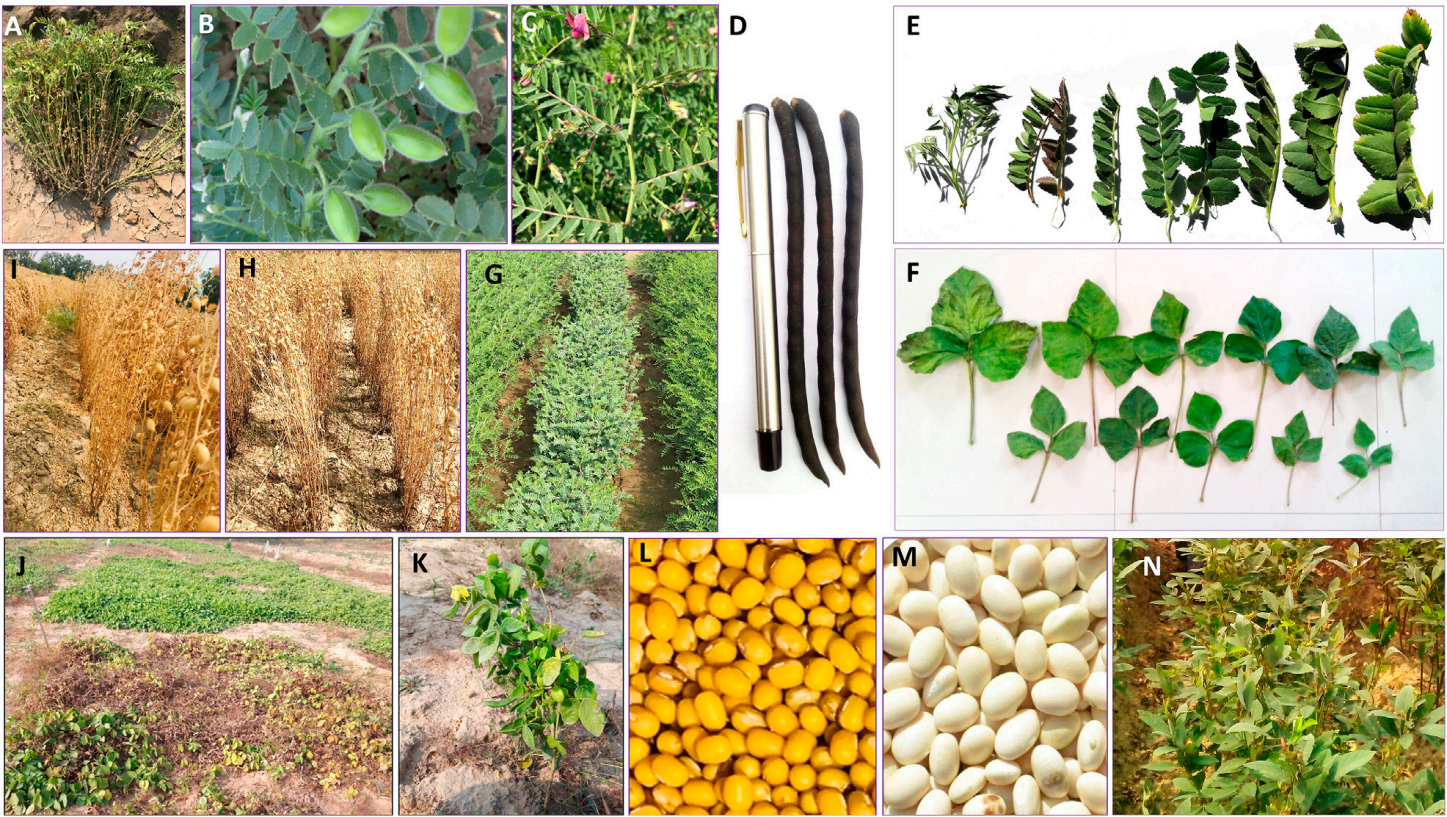
Highlights of various important agro-morphological variations. Genotype (ICC16358) with a large number of branches per plant **(A)**; genotype (IC486088) having upright peduncle and pods **(B)**; genotype (ICC15559) with two to three flowers/peduncle **(C)**; genotype (EC398937) with greater pod length (>15 cm) and a higher number of seeds/pod **(D)**; leaflet size variation **(E)**; leaf size variation **(F)**; genotype with short internode length and compact phenotype **(G)**; genotype (IC24417) with erect and tall growth habit **(H)**; an erect genotype (NBeG 47) in chickpea **(I)**; early-maturing (IC347181) **(J)** and erect type (VLG 39) **(K)** genotypes of horse gram; sona mung with bright yellow seeds having superior visual appeal **(L)** in green gram; a common bean germplasm (IC341862) having pea-shaped, bright white-colored seed with superior visual appeal **(M)**; and a pigeon pea genotype with determinate growth habit **(N)**.

Development of the first core collection in the domain of legumes was reported by Hannan et al. (1994) with the objective of making use of chickpea collections. In this study, a diverse set of 505 chickpea accessions was designated as a core set; this was derived from 7,613 accessions conserved in the Western Regional Plant Introduction Station (WRPIS), USDA. Later, the International Crops Research Institute for the Semi-Arid Tropics (ICRISAT) developed a core set of 1,956 accessions based on information on the geographic origins and on 13 morphological traits for 16,991 accessions (Upadhyay and Ortiz, 2001). Following this exercise, Upadhyay et al. (2007a) identified 28 early-maturing chickpea germplasm lines having wide geographical distribution. Based on multi-location trials of the core set, ICC 16641, ICC 16644, ICC 11040, ICC 11180, and ICC 12424 were further identified as extra-early maturing lines, while ICC 14648, ICC 16641, and ICC 16644 were identified as having higher seed weight. Additionally, in an evaluation of 1,956 accessions of the chickpea core set on 14 agronomic traits, several superior accessions were identified in terms of early flowering, pods/plant, seed yield, and seed weight (Upadhyaya et al., 2007b). Furthermore, in order to reduce the size of the core collections, a mini-core set of 211 accessions was developed based on more extensive phenotypic data and a suitable statistical approach (Upadhyaya and Ortiz, 2001); this has been extensively utilized for the evaluation and identification of important traits (Upadhyaya et al., 2010). Promising accessions for traits such as water use efficiency (ICC 16374, ICC 1422, ICC 4958, ICC 10945, ICC 16374, ICC 16903) and biotic-abiotic stresses were identified (Upadhyaya et al., 2010). Erect type chickpea lines suitable for mechanical harvesting were also identified (Upadhyaya et al., 2017). A similar approach was followed at the Indian National Gene Bank to accelerate the utilization of chickpea germplasm; there, the gene bank’s entire chickpea collection (14,651 accessions) was characterized and evaluated for agronomic traits in 2012, and several promising accessions in terms of agronomic traits were identified (Archak et al., 2016). The characterization of a large number of accessions also provides the opportunity to identify rare and unique morphotypes, which sometimes turns out to be very useful. For example, in the study carried out by Archak et al. (2016), accession IC486088 was found to have upright podding behavior, which makes it a potential donor that could be used in altering chickpea plant type (Singh et al., 2013) (Figure 3). To enhance the utilization of such unique germplasm of economic or scientific value in crop improvement programs, these are registered with a national germplasm registration facility, i.e., the Germplasm Registration and Information System (GRIS; http://www.nbpgr.ernet.in:8080/registration/AboutUs.aspx). As of December 2022, a total of 28 unique accessions of chickpea have been registered by this facility. These unique traits help in the development of plant types and/or high-yielding cultivars. For example, a unique determinate phenotype was identified in BGD 9971; this is considered an important trait in the alteration of chickpea plant type (Hegde, 2011; Ambika et al., 2021). In the F2 line of an inter-specific cross, ICC 5783 (*C. arietinum*) × ICCW 9 (*C. reticulatum*), 3 to 9 flowers per flowering node were observed; this is an important trait for improving chickpea plant type and yield (Gaur and Gour, 2002). Finally, to reduce harvesting and threshing time and cost, chickpea genotypes with the erect plant type were identified and are being used to develop chickpea cultivars suitable for mechanical harvesting (Vishnu et al., 2020). GBM 2 and NBeG 47 (Dheera) are the first two such chickpea varieties that have been released (http://dpd.gov.in/Varieties/Chickpea%20varieties.pdf) (Figure 3).

#### 3.1.2 Biotic stress

**FIGURE 4:**
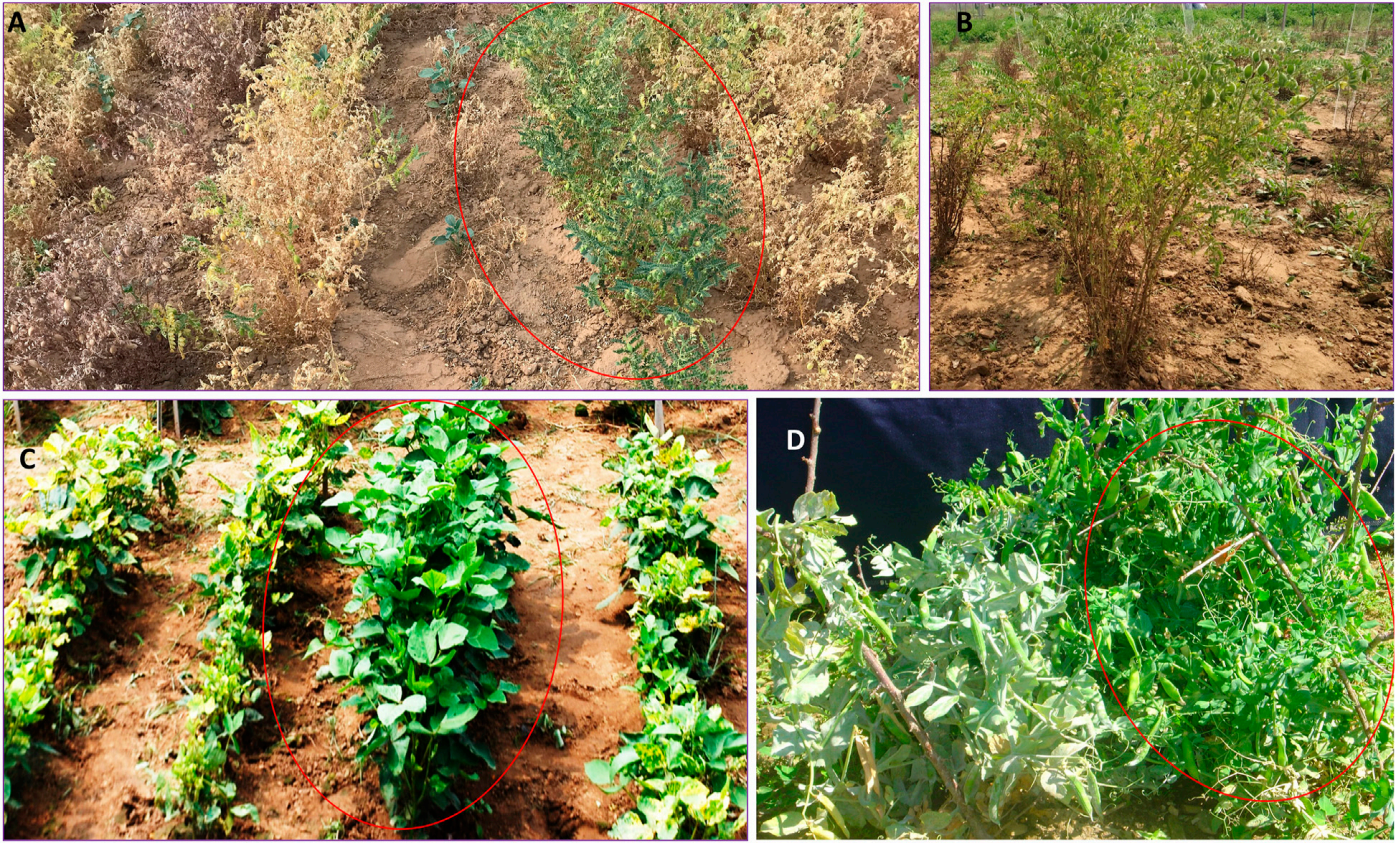
Several promising newly identified resistance donors: IC486215 **(A)** for resistance against dry root rot in chickpea; IC275447 **(B)** for resistance against ascochyta blight in chickpea; IC118998 **(C)** for yellow mosaic disease (YMD) resistance in green gram; and IC278261 **(D)** for powdery mildew resistance in field pea.

In chickpea, the major diseases are fusarium wilt (*Fusarium oxysporum* f. sp. *ciceris*), ascochyta blight (*Ascochyta rabiei* (Pass.) Lab.), collar rot (*Sclerotium rolfsii* Sacc.), dry root rot (*Rhizoctonia bataticola* (Taub.) Butler), and botrytis gray mold (*Botrytis cinerea* Pers. Ex. Fr.), and the major pest is pod borer (*Helicoverpa armigera* Hubner). Chickpea germplasm screening programs have identified rather plentiful instances of donor germplasm resistant to fusarium wilt. However, robust resistant donor sources for dry root rot, botrytis gray mold, collar rot, and pod borer are lacking; thus, germplasm use could result in the identification of moderately resistant donors for these diseases (Pandey et al., 2004; Sharma M et al., 2015; Reddy et al., 2016). The ICAR–National Bureau of Plant Genetic Resources (ICAR-NBPGR) has evaluated over 2,500 accessions for resistance to botrytis gray mold, collar rot, and dry root rot under artificial inoculation and field conditions, but only a few moderately resistant accessions have been identified, such as IC244185, IC251727, ICC6881, and IC350842 for BGM; IC270930, IC95064, IC350829, IC95100, IC209375, IC83805, IC487359, and IC83991 for collar rot; and IC413984, IC397375, IC487359, IC506915, and ICC4295 for dry root rot (unpublished data). This is a sign of the narrow genetic base of the cultivated germplasm. Similarly, no resistance sources have yet been identified for pod borer. Gayacharan U. et al. (2020) have identified several robust resistance sources (viz., IC275447, IC117744, EC267301, IC248147, and EC220109) for ascochyta blight disease using the sick plot method following artificial inoculation in multiple environments and seasons; these are now being utilized in national chickpea breeding programs. Pande et al. (2006) have also identified several other promising chickpea accessions (*viz.*, ICC 17211, IG 69986, IG 70030, IG 70037, and IG 70038), which have shown combined tolerance against ascochyta blight and botrytis gray mold diseases. Finally, Singh (1997) has listed several of the important sources identified at the International Center for Agricultural Research in the Dry Areas (ICARDA) and at the ICRISAT. Various important sources of biotic stress resistance are listed by Singh et al. (2022) and are also presented in Table 4 and Figure 4.

#### 3.1.3 Abiotic stress

In chickpea, the major abiotic stresses are terminal drought, terminal heat stress, and low temperatures during the late vegetative stage. The Northern Plains of India, which was once the most favorable zone for chickpea production, has faced a drastic decline in production of this crop due to a sharp rise in the minimum night temperature (Basu et al., 2009). Terminal heat stress is also a major challenge in the expansion of chickpea cultivation to rice-fallow land, of which there are around 11.7 million ha in the country (Singh N et al., 201a). Therefore, in order to tackle this problem, new sources of tolerance are being sought to enable the development of short-duration and heat-tolerant varieties. Basu and coworkers (2009) screened chickpea germplasm and identified several highly heat-tolerant chickpea lines, *viz.*, ICCV 92944 (JG14), ICCV 37, ICC67, JKG 1, GCP 101, and PG 12. A reference collection for heat stress tolerance has also been developed and screened at the reproductive stage (Krishnamurthy et al., 2011). The researchers observed broad genetic variation in heat-responsive traits, and later identified 10 heat stress tolerance lines under field conditions (Devasirvatham et al., 2015). A mini-core collection has also been screened for resistance to drought stress; five highly tolerant accessions (namely, ICC867, ICC 1923, ICC9586, ICC12947, and ICC14778) were identified (Krishnamurthy, et al., 2010). A germplasm line (ICC4958) developed by Saxena et al. (1993) has been extensively used for breeding drought-tolerant varieties. In-depth molecular analysis of the ICC4958 line has identified a QTL-hotspot region that harbors several traits related to drought tolerance (Bharadwaj et al., 2021). Certain other drought stress tolerant lines have been identified under field conditions (Ganjeali et al., 2011). A mini-core set was also screened for salinity stress resistance under pot conditions and artificial application of saline water (100 mM), which led to the identification of 10 highly tolerant accessions (Serraj et al., 2004). Genotypes ICCV 00104, ICCV 06101, CSG8962, and JG62 have also been identified as promising in terms of salinity tolerance (Kumar et al., 2016). Additionally, a total of 3,276 germplasm lines of chickpea were evaluated against cold stress at the ICARDA, Tel Hadya, Syria, between 1981 and 1987; 21 lines were found to be tolerant of cold stress (Singh et al., 1989). Choudhary and coworkers (2018) list several popular donors that represent the major sources for improvement of chickpea tolerance to abiotic stress. An extensive list of chickpea germplasm lines that have been identified as promising in relation to various abiotic stresses is also given in Table 5.

#### 3.1.4 Nutritional Quality

The chickpea is well known for its nutritionally rich grains, which are widely used as an alternative source of supplementary nutrients. Chickpea grains contain 63% total carbohydrate, 21% protein, and 2.70%–6.48% total fat (Wang et al., 2021). The prominent minerals are K (1.2 g/100 g in desi type, 1.1 g/100 g in Kabuli type), P (0.38 g/100 g in desi, 0.5 g/100 g in Kabuli), Mg (169 mg/100 g in desi, 178 mg/100 g in Kabuli), and Ca (162 mg/100 g in desi, 107 mg/100 g in Kabuli; Wang et al., 2021). Chickpea grains are also a good source of vitamins C, B2, B3, B5, γ–tocopherol, E (α–tocopherol), and folic acid.

Large-scale nutritional profiling has not yet been carried out for legumes, primarily due to a lack of high-throughput nutritional profiling platforms. However, nutrient-specific donors with high mineral content have been identified, such as for Zn (MG–13, MG–17), Ca (PI518255, PI358934), and P (PI339154), and these can be used for biofortification of modern chickpea cultivars (Constantini et al., 2021). In an analysis of 79 accessions, one (LEGCA728) was identified as having high lutein content (28.32 μg g^−1^), and distinct morphotypes were identified as superior in terms of high concentration of specific nutrients (Serrano et al., 2017). In this study, it was observed that nutritional variation is associated with seed morphology. Black and brown seeded varieties were found to have higher dietary fiber content, ranging from 18.0 to 22.1 g 100 g^−1^, and higher polyunsaturated fatty acid (PUFA) content (67.0 g 100 g^−1^ of total fatty acids; Summo et al., 2019). Accessions with brown coloring also have high water absorption capacity (1.9 g water g^−1^ of flour), which makes these varieties suitable for mixing with cereal flours to produce nutritionally rich cereal-based food products. The vitamins, minerals, and fibers present in chickpea grains promote their utilization for many health benefits. Finally, carotenoid concentration (with the exception of lycopene) has been found to be higher in wild germplasm as compared to cultivated types (Jukanti et al., 2012).

### 3.2 Lentil

#### 3.2.1 Agronomic traits

Lentil is one of the eight founder crops of agriculture (Ambika et al., 2022) and the most nutritious cool season legume cultivated in many farming systems worldwide. Lentil is divided into two categories based on seed size, i.e., microsperma (seed diameter 2–6 mm) and macrosperma (seed diameter 6–9 mm), with 100 seed weight ranging from 1.5 to 8.0 g. In order to identify new germplasm for various agro-morphological traits, extensive germplasm exploration, characterization, and evaluation programs have been undertaken globally. As a result, several trait-specific donors have been identified and used to develop improved varieties. High biomass, good plant standing, higher seed weight, and number of pods/peduncle are considered to be crucial traits for yield enhancement in lentil. With the availability of such genotypes having tall (>30 cm), erect growth habits and good standing ability with good ground clearance (>15 cm), high-yielding varieties that are suitable for mechanical harvesting have been developed, such as ILL590, ILL1005, ILL6037, ILL6212, ILL6994, ILL7155, and ILL7947 (Sarker and Erskine, 2006; Kumar et al., 2013). Germplasm lines for early flowering (IC560333, IC559639, IC560111, and IC560148), high biomass (IC559744, IC559608, IC559767, and IC560040), a large number of primary branches (IC559870, IC318881, IC398688, and IC560182), and high yield (IC398094, IC560212, IC560332, and IC560206) have also been identified through gene bank germplasm characterization (Gautam et al., 2013). Mishra et al. (2022b) identified PMF-1, PMF-2, PMF-3, and PMF-4 as producing multiple flowers per peduncle, which is an important trait in lentil breeding. The GRIS portal indicates the registration of accessions for a range of important unique traits, such as extended funiculus, which helps with rapid water uptake (IC317520); multiple flowers and pods per peduncle (IC241473); early flowering and maturity (IC241532); and extra bold seeds (EC499760). A core set of 287 accessions was developed for lentil using diversity documentation on 3,068 accessions conserved at the WRPIS, USDA, by Simon and Hannan (1995). Promising germplasm lines have been identified for various agronomic traits, such as seedling vigor, earliness, number of pods/peduncle, number of pods/plant, and seed weight (Singh., 1995). In another large-scale characterization conducted at the ICAR–NBPGR, accessions were characterized on 26 agro-morphological traits, and a core set of 170 accessions was developed (Tripathi et al., 2021a). Kumar et al. (2013) have also highlighted important lines for traits such as winter hardiness, short duration type, mechanical harvesting, and higher seed weight.

#### 3.2.2 Biotic stress

The major diseases in the lentil crop are fusarium wilt (*Fusarium oxysporum* f. sp. *lentis* (Fol)), root rot complex, rust (*Uromyces viciae-fabae*), stemphylium blight (*Stemphylium botryosum*), powdery mildew (*Erysiphe* spp.), and ascochyta blight (*Ascochyta lentis*); the major pests are pod borer (*Etiella zinkenella*), aphids (*Aphis craccivora*), and seed weevil (*Bruchus spp*.). Several studies have been conducted to identify resistant donors, some of which are listed in Table 4. For example, 12 accessions were identified as resistant to fusarium wilt out of 196 landraces screened under both field and controlled conditions (Pouralibaba et a., 2015). In another study, 93 accessions were screened under three different screening conditions (specifically, a hotspot location, field sick plot, and artificial greenhouse conditions) for resistance to wilt, and two highly resistant germplasm lines (viz., IG 69549 and IG 70238) were identified (Meena et al., 2017). Sources for fusarium disease resistance, such as ILL5883, ILL5588, ILL4400, and ILL590, and for resistance to other important diseases, such as rust (ILL358, ILL4605, ILL5604, ILL6002, and ILL6209), ascochyta blight (Indianhead, ILL358, ILL857, ILL5562, ILL5588, ILL5684, ILL5883, and ILL6024), and Stemphylium blight (ILL 4605), have been highlighted by Kumar et al. (2013). Additionally, 4 lines (RR–107, ILL7207, ILL7716, and ILL7618) have been identified as resistant to rust (*Uromyces fabae* (Pers.) de Bary) out of 286 accessions screened under controlled conditions (Rubiales et al., 2013). Blight (*Stemphylium botryosum* Wallr.) resistance has also been identified in wild lentil germplasm (Podder et al., 2013). Seed weevil (*Bruchus spp*.) is another major threat to lentil grains; therefore, 571 accessions of lentil originating from 27 different countries were evaluated under natural field conditions in central Spain, with wide variation (0%–70%) being observed in infestation rate in the lentil germplasm (Laserna–Ruiz et al., 2012). In this study, a total of 32 accessions with lower levels of infestation were identified. In a separate screening of 300 lentil accessions against root-knot nematode (*Meloidogyne incognita*), 9 accessions were identified as tolerant (Khan et al., 2017; Table 4). Furthermore, these have been registered with the GRIS portal to enhance the utilization of such important sources in lentil breeding programs. Examples of such accessions include IC296883 for multiple resistance against *Meloidoyne incognita*, *M. javanica*, Botrytis gray mold, and pod borer; IC567650 for rust resistance; and IC559673 and IC559890 for nematode resistance.

#### 3.2.3 Abiotic stress

Drought, heat, cold, frost, salinity, and waterlogging are the major abiotic stresses affecting lentil cultivation around the world. Several studies have been conducted to identify germplasm tolerant to these stresses (Table 5). In one such study, 166 lentil accessions were screened under field conditions, and six lines (ILL 7835, ILL 6075, ILL 6362, ILL 7814, ILL 7835, and ILL 7804) were identified for combined heat and drought stress tolerance (Rajendran et al., 2020). The Focused Identification of Germplasm Strategy (FIGS) was used to select 162 accessions for screening against heat and combined heat–drought stresses under field conditions at two locations (El Haddad et al., 2020); one germplasm line (IC621470) has been registered for drought tolerance on the GRIS portal. Based on a salt stress tolerance index, several promising accessions (ILL 5845, ILL 6451, ILL6788, ILL 6793, and ILL 6796) were identified in a screening of 133 accessions under artificial conditions (Ashraf and Waheed, 1990). Accessions ILL52, ILL465, ILL 1878, ILL 1918, ILL7115, ILL7155, ILL468, ILL590, ILL662, ILL669, ILL780, ILL857, ILL975, WA8649041, and WA8649090 have been selected for winter hardiness (Erskine et al., 1981; Hamdi et al., 1996). Additionally, Stoddard et al. (2006) have identified ILL5865 and ILL1878 as lines with good levels of tolerance to freezing. In Australia, in order to expand lentil cultivation, 310 accessions were screened in soils with a high boron concentration; accessions originating from Afghanistan and Ethiopia were found to perform comparatively well under these conditions. Boron-tolerant accessions ILL213A and ILL2024 were also recorded as having higher biomass than boron-intolerant accessions (Hobson et al., 2006).

#### 3.2.4 Nutritional quality

Lentil has a high protein content (20%–27%; Zaccardelli et al., 2012) and contains 2%–3% fat, 50%–65% starch, and 8%–9% soluble sugars (Jood et al., 1998). Lentil protein is considered to be among the most beneficial, as it has good Leu/Ile and Leu/Lys ratios (1.24–1.98 and 1.08–2.03, respectively), high digestibility (∼83%), and strong potential for use in food products (Jarpa-Parra, 2018). Among pulses, lentil is also one of the richest sources of Zn and Fe. A screening of over 2000 cultivated and wild germplasm has revealed a wide range of variation in Fe (42–168 ppm) and Zn (22–101 ppm; Mehra et al., 2018), with accessions EC78933 and EC 78414 found to have particularly high Fe and Zn content, respectively. In one of the experiments conducted by Kumar et al. (2014), 41 genotypes were examined for stability of Zn and Fe content over three locations; L 4704 (136.91 mg/kg grain) and VL 141 (81.542 mg/kg grain) were found to be promising in relation to Fe and Zn, respectively. A germplasm line (IC317520) with an extended funicle has also been identified; this is expected to be associated with shorter cooking time (Tripathi et al., 2021b). Several genotypes have been registered with GRIS: IC208329 and IC208326 for high protein content (27.4%–28.5%), and IC0616579 for high iron 136.91 (mg/kg grain) and zinc (71.69 mg/kg grain) content.

### 3.3 Common bean

#### 3.3.1 Agronomic traits

Common bean is an economically important legume and is cultivated worldwide. In order to assess phenotypic variability in the *ex-situ* collections of the Indian National Gene Bank, 4,274 accessions were characterized on 22 traits, and a good range of variation was observed in leaf length, leaf width, pod length, number of pods per plant, number of seeds per pod, and seed weight (Rana et al., 2015). Promising accessions were identified for early flowering (IC370764), pod length (IC328871, EC271552), pods/plant (EC500299), early maturity (EC0944456), a large number of seeds/pod (IC383008), *etc.* In another study, 203 accessions of a core collection were examined for seed quality traits and to identify promising germplasm lines (Saba et al., 2016). The Andean Diversity Panel (ADP), a regional core collection comprising 396 accessions, with the majority originating from the Andean region, was established in order to enhance germplasm utilization in the region’s common bean improvement program (Cichy et al., 2015a). The ADP consists primarily of popular cultivars, breeding lines, and landraces. The CIAT gene bank conserves over 40,000 common bean accessions, making it the largest collection in the world. In an evaluation of a core set of 1,414 accessions, Amirul et al. (2006) observed wide variability in their morphological, biochemical, and nutritional traits. Through 12 multi-environment trials, a recent study has also identified four specific germplasm from 481 breeding lines with notable agronomic traits; the authors also developed a model to predict genotypic performance under different environmental conditions (Keller et al., 2020).

#### 3.3.2 Biotic stress

The common bean is affected by many bacterial, fungal, and viral diseases, as well as insect pests. Several studies have been conducted to identify resistant sources in common bean germplasm (Table 4). A recent study has identified 14 accessions resistant to angular leaf spot (*Phaeoisariopsis griseola*) under field conditions (Rezene and Mekonin, 2019). Peña et al. (2013) screened 274 germplasm lines under artificial and pot conditions and identified two lines (PI 310668 and PI 533249) showing resistance against damping-off disease (*Rhizoctonia solani*). A set of 248 accessions of wild bean (*Phaseolus* spp.) were screened under greenhouse, pot, and artificial conditions against fusarium root rot (*Fusarium cuneirostrum*) and fusarium wilt (*Fusarium oxysporum*), resulting in the identification of 21 and 16 lines resistant to fusarium root rot and fusarium wilt, respectively (Haus et al., 2021). Urrea and Harveson (2014) carried out screening of 467 germplasm lines against bacterial wilt (*Curtobacterium flaccumfaciens pv. flaccumfaciens*) under pot and artificial conditions, and identified PI 325691 as a resistant line to the disease. The GRIS portal also indicates that several accessions have been registered as resistant to important diseases, such as anthracnose (IC0341862, IC635031, and IC635032), white mold disease (EC271515 and IC278744), and bean common mosaic virus (IC340947 and IC0360831).

#### 3.3.3 Abiotic stress

The common bean is severely affected by abiotic stresses, such as cold, drought, heat, and salinity, and not a great deal of work has been carried out to identify trait-specific donors, with a few exceptions. Urrea and Porch (2009) screened 277 accessions of *P. vulgaris* and *P. acutifolius* under terminal drought stress conditions at Mitchell. The G35346 line has been identified for aluminum (Al) tolerance and used to transfer Al tolerance to common bean varieties (Butare et al., 2012). Tepary bean (*P. acutifolius* Gray), a relative of common bean, is known to have comparatively better tolerance for drought and sub-zero temperatures; on this basis and through preliminary screening of tepary bean accessions, one accession (W6 15578) has been identified as a potential donor for tolerance of both these stresses (Souter et al., 2017). Additionally, Dasgan and Koc (2009) screened 64 lines at 125 mM NaCl to identify salt-tolerant donor lines; a good level of variation was observed, and five highly tolerant genotypes were identified: Yalova 5, TR68587, Kibris Amerikan, Magnum, and Yerhammadisi.

#### 3.3.4 Nutritional quality

Common bean is an excellent source of protein, dietary fiber, vitamins, and minerals. Its grains are a rich source of water-soluble vitamins, particularly thiamin, riboflavin, niacin, and folic acid. Analysis of a Chilean bean core collection of 246 accessions revealed protein content ranging from 183.5 to 259.7 g kg^−1^, Fe content from 68.9 to 152.4 mg kg^−1^, and Zn content from 27.9 to 40.7 mg kg^−1^ (Paredes et al., 2009). Kaur et al. (2009) also studied the physicochemical, hydration, textural, and cooking properties of common bean, observing a wide range of variation in terms of seed density (0.51–2.15 g/ml), hydration capacity (0.03–0.62 g/seed), hydration index (0.16–0.97), swelling capacity (1.24–1.93 ml/seed), cooking time (50–120 min), and amylose content (0.09%–5.02%). Another study revealed the ranges of variation in common bean for antioxidant activity (5.5%–44.9%), starch content (17.4%–40.7%), size of starch granules (1.64–176 μm), rapidly digestible starch (11.1%–19.5%), slowly digestible starch (8.5%–17.3%), and resistant starch (63.9%–76.1%; Sharma et al., 2015). Common bean is well known for rich diversity in seed coat color, and this color plays a major role in the selection, taste, and palatability of particular genotypes. Therefore, to investigate the relationship between color and protein and mineral content, a study was conducted in 100 genotypes having carioca, black, and other grain color patterns (Silva et al., 2012). The results indicated that black-colored beans are richer in protein, iron, and zinc; carioca grains are richer in manganese and magnesium; and grains of other colors are rich in calcium. Ciat-A-257, Bolinha, Iapar 81, Linea 29, and Roxo PV were found to be rich in protein (28.95%–30.40%). Additionally, 206 accessions from the Andean Diversity Panel were evaluated on cooking time, and five accessions (ADP0367, ADP0521, ADP0469, ADP0518, and ADP0452) were identified as promising in terms of shorter cooking time (Cichy et al., 2015b). Germplasm was also compared on nutritional composition and cooking characteristics with its closely related cultivated species, the tepary bean (*Phaseolus acutifolius*), in order to identify superior donors, as the latter species is highly tolerant to abiotic stresses (Porch et al., 2017). The results of this study indicated that there were no species-level differences on most nutritional parameters, with the exception of shorter cooking times for tepary bean accessions (Porch et al., 2017).

### 3.4 Pigeon pea

#### 3.4.1 Agronomic traits

Pigeon pea is a legume of Indian origin (Ambika et al., 2022), and India remains its largest producer and consumer (Bohra et al., 2012). Pigeon pea is a resource-rich crop in terms of genetic and genomic resources, whole genome sequencing information, availability of trait-specific germplasm, genetic stocks, *etc.* The largest collection of pigeon pea germplasm is currently conserved at the ICRISAT gene bank (13,632 acc.), followed by the Indian National Gene Bank, ICAR–NBPGR (11,210 acc.); these collections are the major resources for trait identification and crop improvement. To enhance germplasm utilization, a set of 1,290 pigeon pea accessions (Reddy et al., 2005) has been developed, followed by a mini-core set of 146 accessions (Upadhyaya et al., 2006) and a composite core set of 1,000 accessions, plus a reference set of the most diverse 300 accessions (Upadhyaya et al., 2011a). An exceptionally good level of phenotypic variation has been observed for traits such as pods/plant, number of racemes, plant height, seed yield/plant, and days to maturity (Reddy et al., 2005). Promising accessions included in the pigeon pea composite core set have been listed for important economic traits, such as early flowering, a large number of pods/plant, seed weight, and yield/plant (Upadhyaya et al., 2011b). A vast amount of variability in flowering period has been observed, and a number of genotypes have been reported to show exceptionally short and long flowering durations. ICPL 90011 is reported to be an extra-short duration genotype with the lowest photoperiod sensitivity (Silim et al., 2007). Diverse trait-specific germplasms have been identified for use as potential sources for improvement programs (Upadhyaya et al., 2007c; Mir et al., 2014; Yohane et al., 2020). As of December 2022, 55 pigeon pea germplasms have been registered with the GRIS portal for a range of unique traits, including genetic male sterility (IC296750), cytoplasmic genetic male sterility (IC471860, IC471861, IC296590, IC296592, IC555904, *etc.*), cytoplasmic male sterility (IC296625, IC296623, *etc.*), fertility restoration (IC296805, IC296806, IC296807, *etc.*), early maturity (IC0587711, IC0587712), open flower (IC0573418, IC0573419, IC0573420), determinate growth habit (IC296589), and several other important traits.

#### 3.4.2 Biotic stress

Pigeon pea production is adversely affected by many insects and diseases, such as wilt (*Fusarium udum* Butler), sterility mosaic virus (PPSMV) disease, phytophthora blight (*Phytophthora drechsleri* f. sp. *cajani*), Gram pod borer (*Helicoverpa armigera*), pod fly [*Melanagromyza obtusa* (Malloch)], and spotted pod borer [*Maruca vitrata* (Geyer)]. In one experiment, Saxena et al. (2002) evaluated 271 accessions under natural field conditions, and found that disease severity scores in pigeon pea germplasm ranged from 3 to 9. Screening against fusarium wilt and sterility mosaic disease (SMD) was carried out under artificial conditions for multiple seasons, resulting in the identification of several resistant accessions, viz., ICP 6739, ICP 8860, ICP 11015, ICP 13304, and ICP 14819 (Sharma et al., 2012). Accession ICPW 94 of the wild species *C. scarabaeoides* has been identified as resistant to all isolates of SMD, and is used in crossing programs (Hema et al., 2014). Earlier similar studies were also conducted using petiole grafting and artificial conditions in search of donors for SMD resistance in pigeon pea (Kulkarni et al., 2003; Sharma et al., 2015). Several sources of resistance for various biotic stresses in pigeon pea are listed in a review by Sultana et al. (2021) and in Table 4.

#### 3.4.3 Abiotic stress

Pigeon pea is considered to be a drought-tolerant crop due to its deep root system and wide range in maturity period, which allows it to fit into a wide range of environments and cropping systems (Choudhary et al., 2011). Major abiotic stresses limiting pigeon pea productivity are waterlogging, drought, low temperatures (<10°C), and photoperiod sensitivity. Through several germplasm evaluation programs in pigeon pea, a number of popular donors have been identified; these are major sources for abiotic stress tolerance (Choudhary et al., 2018). In one study, 96 pigeon pea accessions were identified for early flowering; these are considered potential sources for the breeding of early-maturing pigeon pea varieties in order to avoid terminal drought and heat stress (Upadhyaya et al., 2011b). Sultana et al. (2013) screened 272 pigeon pea lines for waterlogging stress tolerance under laboratory and field conditions, and identified 12 lines tolerant to waterlogging. Similarly, in another study conducted under pot conditions for multiple seasons, 24 pigeon pea accessions were identified as waterlogging-tolerant (Krishnamurthy et al., 2012). Various other sources for resistance to important abiotic stresses are also listed in a review by Sultana et al. (2021) and in Table 5.

#### 3.4.4 Nutritional quality

Pigeon pea contains approximately 86.6%–88.0% dry matter, 19.0%–21.7% crude protein, 1.2%–1.3% crude fat, 9.8%–13.0% crude fiber, and 3.9%–4.3% ash content (Amarteifio et al., 2002). Pigeon pea mineral content (mg/100 g dry matter) ranges are as follows: 1845–1941 K, 163–293 P, 120–167 Ca, 113–127 Mg, 11.3–12.0 Na, 7.2–8.2 Zn, 2.5–4.7 Fe, and 1.6–1.8 Cu. However, these values vary with genotype and across different studies (Talari and Shakappa, 2018). Biochemical evaluation of a total of 55 genotypes comprising advanced lines, improved cultivars, and landraces resulted in the identification of variation in four parameters: crude protein content (16.7%–28.4%), total phenol (21.9–84.4 mg/100 g), total flavonoid (16.4–33.4 mg/100 g), and total antioxidant activity (19.2–82.5 mg/100 g) (Cheboi et al., 2019).

### 3.5 Field pea

#### 3.5.1 Agronomic traits

Field pea is cultivated in over 100 countries for fresh and dry grains and for fodder. Over 31,000 germplasm accessions of *Pisum* are conserved *ex situ* in various gene banks, including the Australian Grains Genebank, Australia; the Western Regional Plant Introduction Station, USDA, United States of America; the Leibniz Institute of Plant Genetics and Crop Plant Research, Germany; and the ICAR–NBPGR, New Delhi, India. Although a limited number of large-scale studies have been conducted on the agro-morphological characterization of field pea and for trait identification, several studies nevertheless indicate a substantial amount of phenotypic variability on qualitative as well as quantitative traits, such as days to 50% flowering, seed weight, plant height, and number of pods/plant (Azmat et al., 2011; Bhuvaneswari et al., 2017). The accessions IPF–5–19, EC 8495, HUDP–15, and DDR–30 have been found to show promise in terms of seed yield (Bhuvaneswari et al., 2017). Singh et al. (2010) evaluated 71 accessions on agronomic performance and seed and flower characteristics, identifying promising accessions in terms of early flowering (IC279013), early maturity (IC394017), a large number of pods/cluster (IC279195), longer pods (IC279013), pods/plant (IC219027), seed yield (IC279082), and seeds/pod (IC394028). Genotypes with five flowers per peduncle (VRPM–901–5) and three flowers per peduncle at multiple flowering nodes have been reported in garden pea, which could be highly useful in field pea improvement (Devi et al., 2018). Several unique trait-specific pea accessions have been registered in the GRIS portal, such as IC296677 (leafletless, dual purpose, and high-yielding), IC296678 (dwarf, leafletless), IC296737 (male sterile line governed by a single gene), IC279125 (bold seed with 50.14 g 100 seed weight), IC0610501 and IC630592 (≥ three pods/peduncle), IC636671 and IC640781 (extra-early flowering), and EC414478 (extended funicle).

#### 3.5.2 Biotic stress

The major biotic stresses affecting field pea are powdery mildew (*Erysiphe pisi*), rust (*Uromyces viciae-fabae*), ascochyta blight (complex of *Ascochyta* spp*.*), white rot (*Sclerotinia sclerotiorum* (Lib) de Bary), wilt (*Fusarium oxysporum* f. sp. *pisi*), root rot (many pathogenic fungi), and collar rot (*Sclerotium rolfsii*). Screening against pea weevil (*Bruchus pisorum* L.) in 602 field pea lines, primarily from the Ethiopian Institute of Biodiversity (EIB), Addis Ababa, Ethiopia, resulted in the identification of four resistant lines: 32454, 235002, 226037, and 32410 (Teshome et al., 2015). Large-scale germplasm screening against powdery mildew disease under natural epiphytotic conditions has also been carried out, with the germplasm lines EC598655, EC598878, EC598704, IC278261, and IC218988 being identified as promising (Rana et al., 2013). Nisar et al. (2006) also reported three germplasm lines (Fallon, PS99102238, and PS0010128) to be highly resistant against powdery mildew.

#### 3.5.3 Abiotic stress

Cold, frost, salinity, and heat stresses are the major sources of abiotic stress in field pea crop production. Many studies have taken up the aim of developing lines tolerant to abiotic stresses. In one such study, five field pea germplasm (ATC 104, ATC 377, ATC 968, ATC 3992, and ATC 4204) were identified as frost-tolerant at the reproductive stage through screening of 84 accessions under controlled environmental conditions (Shafiq et al., 2012). Screening of 3,672 pea germplasm lines under field conditions led to the identification of 214 cold-tolerant lines (Zhang et al., 2016). Additionally, 780 accessions were screened for salinity stress tolerance under artificial conditions (Leonforte et al., 2013). Finally, in a recent study, IPFD 11–5, Pant P–72, P–1544–1, and HUDP 11 were identified as heat-tolerant lines based on evaluation under timely- and late-sown field conditions (Lamichaney et al., 2021).

#### 3.5.4 Nutritional quality

Field peas in general have lower protein content (∼25%), very low fat content (∼0.1%), and very high carbohydrate content (∼70%). Major yield-attributing traits in field pea are pods/plant, number of grains/pod, and seed weight. In one study, 94 pea genotypes were examined for pea carotenoid content; higher carotenoid content (10–27 μg/g) was observed in accessions with green cotyledons, and comparatively low carotenoid content (5–17 μg/g) in accessions with yellow cotyledons (Ashokkumar et al., 2015). Pea grains have comparatively higher antioxidant activity than chickpeas. Promising field pea accessions have also been identified in terms of mineral content, such as Zn (IG52442, IG134828), Cu (IG116297, IG52442) and Ca (IG51520, IG52442), by Costantini et al. (2021). Additionally; Singh et al. (2010) have identified lines with shorter cooking time (IC260344) and observed that the genotypes that absorb more water and swell more during soaking require less cooking time. The authors have also identified IC320964 as superior in terms of ash content (3.73%), and several other accessions as promising in terms of their physicochemical properties. In a nutritional analysis of 96 accessions from diverse collections at the USDA National Germplasm Center, Pullman, WA, a wide range of variation was observed in mineral micronutrient content (Hacisalihoglu et al., 2021). An atypical morphotype having extended funicle (EC0414478) was identified in pea germplasm, and this accession was found to be associated with faster water uptake in comparison to the checks included (Tripathi et al., 2021b); this is likely to help with the development of pea cultivars with shorter cooking times.

### 3.6 Cowpea

#### 3.6.1 Agronomic traits

Cowpea is a multi-purpose grain legume (yielding grains, green pods, and leaves) and is widely cultivated in Asia, Africa, and America. It is considered to be one of the best suited crops for hotter, semi-arid agro-climatic conditions, as it requires less water and also grows well in sandy soils. The germplasm conserved in various gene banks has exhibited a good amount of genetic variability, which enables it to grow in various agro-climatic regions and in various soil types. To enhance the utilization of cowpea germplasm, over 12,000 accessions of cowpea were characterized on 28 agro–botanical descriptors at the International Institute of Tropical Agriculture (IITA), Ibadan, and a core set of 2,062 accessions was developed (Mahalakshmi et al., 2007). In another study, 4,000 accessions were characterized in multi-location trials by the ICAR-National Bureau of Plant Genetic Resources (unpublished records). A great deal of variability was observed in plant and seed morphology. Gerrano et al. (2015) identified germplasm lines having desirable grain yield characteristics, such as Fahari, IT93K129-4, Glenda, and vegetable cowpea dakama cream; Nkhoma et al. (2020) identified lines Bubebe, CP411, CP421, CP645, Chimponogo, and MS1–8–1-4 as high-yielding and genetically divergent among 90 genotypes studied, making them ideal parental lines. Cowpea genotypes IT96D-604, 93K-619-1, IT97K-569-9, and IT99K-1060 have also been identified as high-yielding (Goa et al., 2022).

#### 3.6.2 Biotic stress

The major diseases affecting cowpea are cowpea mosaic virus (CpMV) disease, *Cercospora* leaf spot (CLS), brown blotch (*Colletotrichum capsici*), and bacterial blight (*Xanthomonas axonopodis* pv. *vignicola*), while the major pests are pod borer, aphids, thrips and bruchids. The severity of these biotic factors varies with agro-climatic zone and growing conditions. Although cowpea is one of the more prominent legume crops and the largest of the Vigna group, it has not received commensurate research attention. As a result, cowpea improvement has suffered from a lack of reliable donors for resistance to many of these biotic factors. Nonetheless, efforts have recently been undertaken in this direction, and several important and promising donors for resistance to a small number of these biotic stresses have been identified; these are listed in Table 4. In a study that aimed to identify resistant donors for aphid (*Aphis craccivora*), cultivated germplasm (105 accessions) and wild germplasm (92 accessions) were screened under greenhouse conditions; only a single accession (TVNu 1,158) was identified as a resistant line (Souleymane et al., 2013). The findings of this study also indicated that both the cultivated and the wild relatives of this crop have poor genetic bases. Boukar et al. (2019) identified 14 lines having resistance to bacterial blight (*Xanthomonas axonopolis* pv. *vignicola*) under artificial inoculation. In another study, 225 germplasm lines were screened against CpMV, CLS, and cowpea rust (*Uromyces vignae*), resulting in the identification of promising accessions for resistance to these pathogens (Deshpand et al., 2010; Table 4). Finally, Tripathi et al. (2020) identified EC528425 and EC528387 as tolerant to bruchid (*Callosobruchus maculatus*) through the screening of 103 cowpea lines using a ‘no-choice’ test method.

#### 3.6.3 Abiotic stress

The major abiotic stresses are drought, heat stresses, and poor soil fertility, especially in sub-Saharan Africa (SSA), where cowpea is grown as a major crop, as well as soil salinity in almost all irrigated areas worldwide (Horn and Shimelis, 2020). Several studies have been conducted to identify resistant donors (see Table 5). Five lines with superior drought stress tolerance (viz., TVu1436, TVu9693, TVu12115, TVu14632, and TVu15055) have been identified using the water withdrawal method under field conditions (Fatokun et al., 2012), while Dagupan Pangasinan, UCR 369, and Negro have been identified as tolerant to waterlogging at the seedling stage (Olorunwa et al., 2022). Accessions EC472250, EC472267, EC472285, EC472286, EC472289, and Pusa Komal have been identified as tolerant to heat stress through screening in multiple seasons under field conditions (Mishra et al., 2005). Accessions PI582422, 09–529, PI293584, and PI582570 have been identified as tolerant to salinity stress under artificial screening conditions through imposition of salinity stress (150 mM NaCl) at the seed germination stage (Ravelombola et al., 2017). Other similar studies have also identified lines tolerant to salt stress using different NaCl concentrations (150 mM and 120 mM) at the germination and seedling stages (Ravelombola et al., 2018; Dong et al., 2019). Based on screening of 155 cowpea lines in 200 mM NaCl, several promising lines were identified as salt tolerant, i.e., PI354686, PI353270, PI354666, PI354842 PI548785, PI582466, PI339599, and 09-697 (Dong et al., 2019).

#### 3.6.4 Nutritional quality

Cowpea is a major source of nutrition in sub-Saharan Africa, Asia, and Latin America. Based on nutritional profiling of 100 breeding lines on a dry weight basis, a significant range of variation has been observed in terms of protein content (22.9%–32.5%), ash content (2.9%–3.9%), fat content (1.4%–2.7%), and carbohydrate content (59.7%–71.6%; Nielsen et al., 1993). Genotypes also vary in 50% cooking time, which ranges from 21.1 to 61.9 min, and promising donors have been identified, such as IT83S–872 for protein content, IT84S–2085 and IT86D–466 for ash content, and IT85F–2805 for shortest cooking time (Nielsen et al., 1993). In a study aiming to investigate nutritional variability in immature cowpea pods, 22 genotypes were analyzed on various nutritional composition parameters; genotypes such as ITOOK-1060, TVU-14196, and 98K–5301 were found to be superior on such parameters as Mg, Na, Mn, Boron, Al, Zn, Cu, K, P, and protein (Gerrano et al., 2017). The fresh young leaves of cowpea are also consumed in several countries; therefore, analyses have been conducted of the nutritional composition of 15 varieties and the sensory attributes of 10 varieties (Ahenkora et al., 1998). In this study, nutrient concentration in cowpea leaves on a dry weight basis was found to range from 303.8 to 468.9 mg/100 g for phosphorus, from 33.5 to 148.0 mg/100 g for ascorbic acid, and from 27.1% to 34.7% for protein.

### 3.7 Black gram

#### 3.7.1 Agronomic traits

Black gram is a grain legume of Indian origin, primarily cultivated in South Asian regions. Although black gram is an important legume, its productivity level is very poor compared to that of other legumes, which can mainly be attributed to a lack of good plant ideotypes and resistance sources for major diseases in its cultivated gene pool (Kumar and Singh, 2014; Shanthi et al., 2019; Subramaniyan et al., 2022). Therefore, to identify donors for desired agro-morphological traits, 484 accessions have been characterized on qualitative and quantitative traits; a good deal of variation was observed in flowering and maturity period and in yield (Ghafoor et al., 2001). Recently, 840 accessions of black gram were also characterized, resulting in the identification of promising germplasm lines in terms of early flowering (IC343936, IC436615), synchronous flowering (IC73523, IC396032, IC485444), pod length (IC438379), number of seeds/pod (IC472051_2, IC565238), and seed weight (IC485605_2, IC485588) (Gayacharan et al., 2022). For novel trait generation in black gram, gamma-irradiated mutants were generated using black gram cultivars ADT 3, Co 6, and TU 17-9, which have exhibited high plant yield (Dhasarathan et al., 2021). Additionally, RBU1012 and Pant U-19 have been found to be the most stable genotypes in terms of yield when evaluated under field conditions (Singh N. P et al., 2016). The GRIS portal indicates that unique germplasms of black gram such as IC296878 (dwarf with ground pod bearing habit), IC553269 (brown pods with yellow seeds), IC594172 (male sterile flowers with protruded stigma and crumpled petals), IC594173 (sympodial pod-bearing habit), IC426765 (photosensitive), and IC636672 (extra-early maturing) have been registered for important traits.

#### 3.7.2 Biotic stress

Urdbean leaf crinkle disease (ULCD) and mungbean yellow mosaic disease (MYMD) are the two major diseases of the black gram crop. Yield losses may reach or exceed 60%, depending on the susceptibility of the host plant, if the crop is affected in its early vegetative stage. Nevertheless, unlike green gram, black gram has a high level of resistance against MYMD in its cultivated gene pool, as revealed in a large-scale evaluation under field and artificial conditions conducted during 2019 and 2020 (unpublished records). Several black gram sources of MYMD resistance, identified on the basis of field screening, are highlighted in a review published by Mishra et al. (2020a). Urdbean leaf crinkle virus (ULCV) disease has spread across all the cropping systems in India, and yield losses can reach 100% if the disease outbreak occurs at the early growth stage under favorable weather and host genotype conditions (Biswas et al., 2009). Resistance sources for ULCV have been reported by several researchers (Ashfaq et al., 2007; Gautam et al., 2016); several such sources for this and other diseases are listed in Table 4. In the GRIS database, accessions IC0570267, IC0570268, IC0570269, IC11613, IC636672, IC0144901, and IC485638 are registered as MYMD resistant, and IC0585931 as bruchid resistant. Powdery mildew (*Erysiphe polygoni*) and *Cercospora* leaf spot (*Cercospora canescens*) are the other major diseases of black gram. The major pests affecting this crop include spotted pod borer (*Maruca testulalis* r), whitefly (*Bemicia tabaci*), bruchids (*Callosobruchus chinensis*. and *C. maculatus*), and nematodes (*Meloodogyne incognita*, *M. javanica*, and *Heterodera cajani*), for which reliable sources of resistance are lacking. Bruchids begin infesting the crop during the pod maturity stage, and they are the cause of up to 90% of produce losses (Soundararajan et al., 2012).

#### 3.7.3 Abiotic stress

The crop is grown in a rainfed environment under tropical and sub-tropical climatic conditions. Therefore, terminal drought and heat, as well as waterlogging, are the major constraints on black gram production. Salinity is another problem, particularly in arid and semi-arid regions. Only a small number of studies have examined the potential for improvement of black gram in terms of resistance to abiotic stresses. Saline-tolerant lines, such as BARI Mash-1 (Hasan et al., 2017), VNBG 017, AUB 3, and AUB 20 (Priyadharshini et al., 2019), have been identified as promising under artificial screening conditions. Under natural waterlogging conditions during a germplasm characterization program, a small number of germplasm lines have been identified as tolerant; these were further evaluated under artificial waterlogging conditions, and accessions IC530491 and IC519330 were found to be tolerant to waterlogging (Bansal et al., 2019). In another study, 26 genotypes were analyzed under waterlogging stress; a large amount of variation was observed in various quantitative traits, and BU Acc 25, BU Acc 17, and BU Acc 24 were identified as the strongest performers in terms of yield (Rana et al., 2019). In terms of drought stress tolerance, cultivars VBN4 and K1 have been identified as promising based on protein and biochemical analyses (Sai and Chidambaranathan, 2019).

#### 3.7.4 Nutritional quality

Black gram grains are a rich source of protein (22%–26%) and moderately high in calories (ca. 350 cal/100 g), carbohydrates (ca. 56.6%), and fat (1.1%–1.2%) (Panhwar, 2005; Suneja et al., 2011). They also contain vitamins, viz., Vit. B1 (0.42 mg/100 g), Vit. B2 (0.37 mg/100 g), Niacin (2 mg/100 g), and minerals, viz., Ca (185 mg/100 g), Fe (8.7 mg/100 g), and P (345 mg/100 g) (Panhwar, 2005). However, a limited amount of germplasm has been nutritionally profiled for the identification of nutrient-rich lines. Black gram is reported to exhibit a substantial amount of variation in nutrient content between the whole grain and its milled fraction (Girish et al., 2012). A small number of genotypes among 26 investigated, such as Shekhar 2, have been found to have high Fe and Zn content, and genotypes Yakubpur, PU 31, IPU 99–200, and PDU 1 have been found to have high polyphenol content (Singh J et al., 2017). There is a need for large-scale nutritional profiling to develop an understanding of nutritional variability in the germplasm and to identify superior genotypes with minimal levels of anti-nutritional factors to enhance the palatability of the crop.

### 3.8 Green gram

#### 3.8.1 Agronomic traits

Green gram is a highly nutritious and palatable grain legume cultivated in Asia, primarily for its grains. Green gram cultivation faces constraints such as a narrow genetic base in the cultivated gene pool, a lack of ideal plant type, and many biotic and abiotic stresses. In order to identify new donors, 1,532 *ex situ* collections of green gram conserved in the Indian National Gene Bank were characterized, potential donors for certain agro-morphological traits were identified, and a core set of 152 accessions was also developed (Bisht et al., 1998). A good level of variation was observed in branch length, nodulation, number of pods bearing a peduncle, number of pods per plant, and yield per plant. The World Vegetable Center, Taiwan, holds over 6,700 accessions of green gram, which have been utilized for the development of a core set of 1,481 accessions based on geographic stratification and clustering of genotypes on eight phenotypic traits (Schafleitner et al., 2015). This core set was genotyped using 20 microsatellite markers, and a mini-core set of 289 accessions was developed; this is now extensively utilized for trait identification. In another large-scale characterization and preliminary evaluation of green gram germplasm, promising germplasm lines were identified in terms of early flowering (EC398944, EC398883), synchronous flowering (EC396115, IC76414, and IC488968), greater pod length (EC398937), seed weight (EC398903, EC398884, and EC396413), *etc.* (Gayacharan K. et al., 2020). Recently, the entire green gram *ex situ* collection of the Indian National Gene Bank has been characterized in multi-location trials, and a diverse core set of 400 accessions has been developed (unpublished records). Photoperiod-insensitive genotypes (EC 318985–319057) have also been identified in green gram (Pratap et al., 2014; Pratap et al., 2019). The GRIS portal lists a number of accessions with unique traits, such as a photosensitive nature (IC546478), high seed weight (IC418452), early maturity (IC0589309, IC589310, IC39289, and IC639796), and penta-foliate leaves (IC296679).

#### 3.8.2 Biotic stress

Green gram production is affected by biotic stresses such as yellow mosaic disease (YMD), pod borers, and storage pests. YMD is a comparatively new disease in green gram and is spreading rapidly into new areas, which is a cause for concern. In YMD-susceptible genotypes of green gram, yield losses up to 85% are reported (Karthikeyan et al., 2014), but it has been observed that losses may reach 100% if the crop is infected at seedling stage. Resistance sources are lacking in the entire cultivated gene pool of the crop, as revealed in a field screening of 4,100 accessions at New Delhi (a YMD hotspot location). However, variability in the severity of the disease is observed according to multiple factors, such as genotypic constitution, vector population load, weather conditions, presence of multiple virus strains, *etc.* (unpublished record). Similar reports have also made by other researchers based on germplasm screening (Shad et al., 2006). Several resistant sources for YMD are listed in a review (Mishra et al., 2020a). There are also several reports of YMD resistance in green gram under field conditions (Iqbal et al., 2011; Mohan et al., 2014; Nainu and Murugan, 2020). Aside from YMD, Duraimurugan et al. (2014) identified four lines (viz. LM 131, V 1123, LM 371, and STY 2633) as resistant against bruchid beetle based on a ‘free choice’ and ‘no choice’ test method. Spotted pod borer (*Maruca vitrata*) also causes severe damage to the crop, if not controlled at the appropriate stage of crop growth, and there are no resistant sources available for this pest. Sandhya et al. (2014) have reported that KM–9–128, KM–9–136, RMG–492, LGG–5, and LGG–538 are tolerant to *Maruca vitrata* following field screening of 110 genotypes.

#### 3.8.3 Abiotic stress

Green gram is primarily grown under rainfed conditions; thus, abiotic stresses such as drought, waterlogging, heat, and salinity affect crop production (Singh and Singh, 2011). In general, reliable tolerant donors for these abiotic stresses are lacking in this crop. Forty-one elite lines were screened for heat stress tolerance under late-sown conditions; of these, five lines (viz., EC693357, EC693358, EC693369, Harsha, and ML1299) showed heat stress tolerance (Sharma et al., 2016). Additionally, IC333090 and IC507340 were found to be drought tolerant, out of 100 lines screened under hydroponics conditions (Meena et al., 2021). Mung bean lines OBGG-2013-9 and OBGG-2013-14 have also been reported to exhibit cold tolerance (Kabi et al., 2017).

#### 3.8.4 Nutritional quality

Green gram is nutrient-rich and possesses various health benefits, such as antioxidant, anti-cancer, anti-inflammatory and hypolipidemic activity (Sudhakaran and Bukkan, 2021). Because of its high nutritional value, green gram is regarded as “green pearl” (Nair et al., 2013). It contains approximately 19.7%–29.1% protein (Bartwal et al., 2022), 61%–63% carbohydrates, 1.1%–2.3% fat, 3.2%–4.2% ash, 0.03–0.06 g Fe kg^−1^, and 0.02–0.04 g Zn kg^−1^ (Nair et al., 2013; Sudhakaran and Bukkan, 2021). The nutritional composition of green gram and black gram is very similar, but green gram is reported to have higher moisture, fat, and protein content (Shaheen et al., 2012). A small number of accessions with particularly high nutritional value are listed in the GRIS portal; these could potentially function as donors for nutritional improvement of the crop. Specifically, accessions IC296771 (27.8%) and IC573456 (25.8%) are registered for high protein content, and IC573449, IC573450, IC573451, IC573453, and IC573454 for high Fe and Zn content.

### 3.9 Horse gram

#### 3.9.1 Agronomic traits

Horse gram is one of the least utilized and least studied legumes. The crop is known for its nutritional and therapeutic value, and is primarily cultivated in hill states and dry areas of southern India. This crop has failed to attract the attention of breeders and researchers due to a lack of ideal ideotypes and morphological variation. A small number of characterization and evaluation studies have been conducted, indicating comparatively wide variation in terms of plant height, pod length, seed test weight, and pods per plant (Singh et al., 2019). Additionally, genotypes CRHG-6 and CRHG-8 are of the non-shattering type, which has been developed through mutation breeding (Salini et al., 2014). In a characterization study examining seven qualitative and quantitative traits in 66 horse gram genotypes, a good amount of variability was observed for pod length and pods per plant (Gomashe et al., 2018). Priyanka et al. (2021) studied 12 quantitative traits across 252 horse gram genotypes, reporting that the highest yield was 65.61 g per plant. Another characterization and evaluation study of 51 accessions led to the identification of several promising accessions (viz., S44/L23, S56/L29, S8/L4, S96/L49, and S29/L14) in terms of early flowering and maturity (Kaundal and Kumar, 2021). In the GRIS portal, only one accession (IC587788) is registered for high fodder yield.

#### 3.9.2 Biotic stress

Horse gram is susceptible to various biotic stresses and still lacks resistant donors for use in crop improvement programs. Only a small number of studies have been carried out to identify resistant donors for selected diseases and pests, such as YMD, wilt, anthracnose, and storage pests. Parimala et al. (2011) identified horse gram accessions AK-38, HG-14, HG-52, HG-59, HG-63, HG-75 as having resistance against horse gram YMD, and AK-38 and HG-46 as resistant to powdery mildew disease. In another study, accessions HG 63, HG 58, HG 50, and Palem 2 were identified as resistant to wilt disease (Durg, 2012). Accession IC470275 has also been identified as resistant to anthracnose disease (Colletotrichum dematium) (Sankar et al., 2015). Finally, horse gram lines Palem-1, Palem-2, AK-21, and NSB-27 have been identified as resistant against Callosobruchus chinensis, a storage pest (Divya et al., 2012; Divya et al., 2013). Accession IC587786 is registered on the GRIS portal as resistant to anthracnose disease.

#### 3.9.3 Abiotic stress

Horse gram germplasm have been screened against abiotic stresses, such as drought, salinity, moisture, and heavy metal stress. Several germplasm lines, such as M-249 and HPK-4, have shown resistance against drought stress (Bhardwaj and Yadav, 2021). Yasin et al. (2014) identified a line tolerant to moisture stress, namely D13. The variety Paiyur-2 was found to have high proline and glycine betaine content and lower lipid peroxidation under salinity stress (Kanagaraj and Sathish, 2017). This genotype was further tested for antioxidant activity, and was found to exhibit enhanced antioxidant activity under salinity stress (Desingh and Kanagaraj, 2019). Separately, the same Paiyur-2 variety was found to be promising for salinity tolerance, and heavy metal tolerance was observed in Madhu (for chromium) and in HGR-4 (for nickel; Dhali et al., 2021; Edulamudi et al., 2021). Based on a screening of 88 germplasm lines for biochemical parameters, accessions TCR491, IC110286, IC56145, and IC53641 were identified as suitable for environments imposing drought stress (Sharma and Chahota, 2022).

#### 3.9.4 Nutritional quality

Horse gram is used as a food and fodder crop and is known for its medicinal and therapeutic uses. It provides protein (17.9%–25.3%), carbohydrates (51.9%–60.9%), lipids (0.58%–2.06%), and vitamins, such as riboflavin, niacin, and vitamin C (Jha et al., 2022). The protein content of horse gram is relatively high compared to that of green gram, black gram, dry peas, the kidney bean, chickpea, or pigeon pea (Longvah et al., 2017). As consumers have become more health conscious, consumption of sprouted horse gram seeds has increased. In the seeds, albumin–globulin is a major contributor (∼75.27–78.76%) to the total protein content. The seeds are low in fatty acid content but rich in dietary fiber, required for proper functioning of the lower intestine (Kawale et al., 2005). Horse gram seeds also exhibit antioxidant activity and radical scavenging activity. In a recent study, metabolic profiling was conducted for 96 accessions of horse gram, which were derived from 700 accessions spread across the entirety of India (Gautam and Chahota, 2022). Tremendous variability in protein content was observed, with the lowest protein content (13%) being found in IC120837 and TCR-1439, while a related wild species (*Macrotyloma sar-gharwalensis*) had the highest protein content (40%). Accessions IC280031 and IC139356 were found to be most nutritive, as the largest number of metabolites (44) was observed for these among the 96 lines selected in an analysis using ^1^H NMR spectroscopy (Gautam and Chahota, 2022). An earlier study had also identified the species *Macrotyloma sar-gharwalensis* (IC212722) as containing 34.88% protein (Yadav et al., 2004).

### 3.10 Moth bean

#### 3.10.1 Agronomic traits

Moth bean is considered to be a hardy crop suitable for hot, arid regions. In addition to this, it also helps to reduce soil erosion, particularly in sandy deserts, due to its extensive root system and profuse foliage cover. Most local cultivars continue to have wild traits, such as pod-shattering, a trailing and spreading growth habit, asynchronous maturity, and a photo-sensitive nature. Few studies have been conducted to explore the existing variability in the gene pool. However, a good amount of phenotypic variability in moth bean was reported by Chaudhary et al. (2021) in an evaluation of 40 genotypes on 10 morphological traits. In another study, accessions IC 36607, IC 39675, IC 251908, IC 36563, and IC 36245 exhibited higher seed yield as compared to checks (Vir and Singh, 2015). Similarly, 50 genotypes of moth bean were phenotyped on 12 quantitative traits and exhibited high levels of variability (Sahoo et al., 2019). Additionally, Singh S et al. (2017) have developed a variety (RMO 257) with superior agronomic traits, such as plant height, dry matter accumulation, and seed yield. A small number of moth bean genotypes are registered in the GRIS portal for early maturity (IC432859 and IC120963) and for single stem formation (IC432859).

#### 3.10.2 Biotic stress

The major biotic stresses are YMD, leaf crinkle disease (LCD), bacterial leaf spot (*Xanthomonas phaseoli*), Cercospora leaf spot (*Cercospora dolichi*), charcoal rot (*Macrophomina phaseolina* Tassi), pod borer, and bruchids. Accessions IC36522 and IC36217 have been identified as YMD resistant as a result of screening under field conditions in multiple seasons (Singh et al., 2020). Meghwal et al. (2015) screened 204 germplasm lines and identified 14 accessions resistant to YMD. Resistance to leaf crinkle virus and *Cercospora* leaf spot has also been reported in the crop (Singh et al., 2020). Vir and Singh (2015) identified LCD resistance in moth bean under field conditions in multiple seasons.

#### 3.10.3 Abiotic stress

Moth bean is one of the best-suited crops for arid and semi-arid environments, and is highly tolerant to drought and heat stress. Although only a small number of moth bean accessions have been investigated, a good amount of variation in the germplasm has been revealed in terms of resistance to drought and heat stress; this could be exploited for crop improvement in order to sustain its productivity amid climate change (Vir and Singh, 2015; Pal et al., 2020). Accessions such as IC129177, IC103016, IC415139, IC 415155, IC36157, Maru moth, and Jadia, which have been identified as tolerant to drought stress, can serve as donors for crop improvement (Malambane and Bhatt, 2014). Additionally, in a separate study, lines IC103016, IC36011, and IC36157 have been identified as promising for drought tolerance (Sachdeva et al., 2016).

## 4 Role of genomics in enhancing grain legume germplasm utilization and attaining higher genetic gains

The first step toward enhancing the utilization of grain legume crop germplasm accessions for trait discovery and subsequent genetic improvement requires thorough and extensive genotypic and phenotypic characterization of such accessions using large-scale data (Rasheed et al., 2017). The numerous germplasm resources (including landraces, wild accessions, cultivated varieties, and breeding lines) available for diverse grain legume crop species, representing diverse agro-climatic regions of the world, have been stored efficiently at various national and international gene banks and repository centers. Considering the difficulties involved in genotypic and phenotypic characterization of this vast set of available germplasm resources, efforts are currently being made to develop core and mini-core collections in the case of several legumes by identifying the greatest amount of genetic diversity that can be represented with a minimal number of accessions (Table 3). This is where the crucial role of genomics comes into play, especially as a means of producing realistic estimates of the level of molecular diversity existing among germplasm accessions, which enables efficient screening of unduplicated authentic accessions in the process of constructing core collections of grain legumes.

Tremendous technological advances made over the last decade in sequencing and other high-throughput sequence- and array-based genotyping assays have supplied much-needed momentum to germplasm characterization. Draft and reference whole genomes, resequencing information, and global transcriptome information have now been decoded for many important grain legume crop plants using first-generation Sanger sequencing and next-generation sequencing (NGS)-based second-generation short read and third-generation long read sequencing assays; the results of these are now publicly accessible (Table 6; Michael and Jackson, 2013; http://www.embl.de; http://www.ebi.ac.uk; http://www.ddbj.nig.ac.jp; http://www.phytozome.org). Grain legumes were once considered to be resource-poor crops, but recent efforts by national and international organizations has altered this trend. As a result, a vast amount of genome sequence information, including whole genome sequences, is available in public databases (Table 6). This sequence information has since been used to understand the genomic features and evolutionary characteristics of the crops in question, and also to develop a vast range of genomic resources, including molecular markers (Garg et al., 2022). Among several sequence-based molecular markers that have been made available, simple sequence repeats (SSRs) and single-nucleotide polymorphisms (SNPs) have occupied a central position, finding extensive use in allelic diversity screening and other genomics-assisted crop improvement programs due to their genome-wide distribution, multi/bi-allelic nature, and amenability to high-throughput detection and genotyping assays (Lateef, 2015). The availability of several high-throughput genotyping platforms and the rapid evolution of the chemical techniques that they employ has enhanced the precision and pace of large-scale mining and genotyping of SSR and SNP markers (Kujur et al., 2015). High-throughput genotyping of SSR and SNP markers in a larger set of germplasm accessions and core or mini-core collections of grain legumes has been expedited *via* the use of various array-based and NGS assays, especially automated fragment analyzer (ABI3730xl automated DNA sequencer), Illumina GoldenGate, and Infinium assays; the Fluidigm dynamic array; KASP (KBioScience Allele-Specific Polymorphism) profiling; MALDI–TOF; the Affymetrix GeneTitan SNP Chip array; and Genotyping-By-Sequencing (GBS) assay (Varshney et al., 2013a; Jain et al., 2013). Among these, the MALDI–TOF, Illumina GoldenGate, and Infinium assays, SNP Chip Array, and KASP profiling have come to be considered highly advantageous and are utilized widely for high-throughput genotyping of previously mined SNP markers in many crop plants (Gaur et al., 2012; Hiremath et al., 2012). In particular, GBS assay has been extensively utilized for simultaneous genome-wide discovery and genotyping of SNPs in diverse plant species (Sonah et al., 2013; Spindel et al., 2013). Its development has thus expedited the mining of novel functional allelic variants and their large-scale validation and genotyping at the whole genome level for efficient trait association mapping of diverse small- and large-genome grain legume crop plants.

**TABLE 6:** Whole genome sequence information available for grain legume crops.

Crop	Project ID	Cultivar	Assembly level	Assembly size (Mb)	Scaffold N50 (Mb)	Sequencing chemistry	Genome coverage %	No. of predicted protein-coding genes	References
Chickpea (*Cicer arietinum*)	ASM33114v1	CDC Frontier	Chromosome	532.29	39.99	Illumina Hiseq 2000	73.8	28,269	Varshney et al. (2013b)
Chickpea (*Cicer arietinum*)	ASM34727v4	ICC4958	Chromosome	511.02	39.90	454; Illumina GAIIx	94	30,257	Parween et al. (2015)
Chickpea (*Cicer reticulatum*)	ASM368901v2	PI489777	Chromosome	416.9	39.84	Illumina HiSeq	78	25,680	Gupta et al. (2017)
Cowpea (*Vigna unguiculata*)	ASM411807v1	IT97K-499-35	Chromosome	519.43	1.64	PacBio; Bionano	91	29,773	Lonardi et al. (2019)
*Asparagus* bean (*Vigna unguiculata* ssp. *sesquipedialis*)	ASM395868v2	Xiabao II	Chromosome	632.8	2.7	Illumina HiSeq 4000	342	42,609	Xia et al. (2019)
Green gram (*Vigna radiata*)	Vradiata_ver6	VC 1973A	Chromosome	431	1.52	Illumina HiSeq2000	80	22,427	Kang et al. (2014)
Green gram (*Vigna radiata*)	SRRS9994113	VC 1973A	Chromosome	476	5.2	PacBio RS II	87.1	30,958	Ha et al. (2021)
Black gram (*Vigna mungo*)	ASM1909614v1	Pant U-31	Scaffold	474	1.42	Illumina HiSeq; Oxford Nanopore GridION	82	42,115	Jegadeesan et al. (2021)
Black gram (*Vigna mungo*)	ASM1342719v1	Chai Nat 80	Chromosome	499	43.17	Illumina HiSeq	92	29,411	Pootakham et al. (2021)
Rice bean (*Vigna umbellata*)	ASM1883591v1	Himshakti	Scaffold	414	0.08	Illumina; PacBio	-	31,276	Kaul et al. (2019)
Pigeon pea (*Cajanus cajan*)	PRJNA72815	ICPL 87119 (Asha)	Chromosome	605.78	0.52	Illumina Hiseq 2000; Sanger sequencing	72.7	46,750	Varshney et al. (2011)
Pigeon pea (*Cajanus cajan*)	PRJNA68667	ICPL 87119 (Asha)	Contig	510.8	-	FLX 454; Illumina HiSeq	75.6	47,004	Singh et al. (2012)
Adzuki bean (*Vigna angularis*)	PRJNA261643	Jingnong 6	Chromosome	450	1.29	Illumina HiSeq 2000	83	34,183	Yang et al. (2015)
Adzuki bean (*Vigna angularis*)	PRJDB3778	Shumari	Chromosome	462	3.0	PacBio RSII; Illumina HiSeq2500	85.6	30,507	Sakai et al. (2015)
Adzuki bean (*Vigna angularis*)	PRJNA253346	Kyungwonpat	Chromosome	443	0.7	Illumina Roche	75	26,857	Kang et al. (2015)
Pea (*Pisum sativum*)	PRJEB31320	Caméor	Chromosome	3920	0.41	Illumina	88	44,756	Kreplak et al. (2019)
Common bean (*Phaseolus vulgaris*)	PRJNA41439	G19833	Chromosome	472.5	50.3	ABI 3730; 454 FLX; Illumina GAII	98	27,197	Schmutz et al. (2014)
Common bean (*Phaseolus vulgaris*)	PRJNA221782	BAT93	Chromosome	458.2	0.43	454; SOLiD; Sanger	81	30,491	Vlasova et al., 2016
*Lima* bean (*Phaseolus lunatus*)	PRJNA596114	G27455	Chromosome	541.5	47.8	PacBio Sequel; Illumina HiSeq		28,326	Garcia et al. (2021)
*Lima* bean (*Phaseolus lunatus*)	PRJNA647124	Bridgeton-DES4	Scaffold	597.4	2.9	Illumina HiSeq	91	64,541	Wisser et al. (2021)
Grass pea (*Lathyrus sativus*)	PRJEB33571	LS007	Scaffold	6200.8	0.06	Illumina; Oxford Nanopore	89.8	33,819	Emmrich et al. (2020)
Horse gram (*Macrotyloma uniflorum*)	PRJDB5374	HPK4	Scaffold	259.2	2.8	Illumina HiSeq 2000; Illumina MiSeq	89	36,105	Shirasawa et al. (2021)
Horse gram (*Macrotyloma uniflorum*)	PRJNA400556	PHG-9	Scaffold	279.12	1.1	Illumina HiSeq; PacBio	83.53	24,521	Mahesh et al. (2021)

Source: NCBI database (https://www.ncbi.nlm.nih.gov/).

Using the aforementioned high-throughput marker-based genotyping strategies, along with large-scale multi-environment phenotyping information, sets of 211, 146, 184, and 289 germplasm accessions belonging to core or mini-core collections of chickpea, pigeon pea, groundnut, and green gram, respectively, have been developed. These have been collated based on the 16,991, 13,632, 15,490, and 6700 accessions available for these respective crop species as a result of the efforts of international institutes such as the IRRI, ICRISAT, USDA, and the World Vegetable Center (Upadhyaya et al., 2001; 2002; Zhang et al., 2011; Schafleitner et al., 2015). These core or mini-core germplasm resources, readily available for many grain legume crop plants, are the primary sources of trait discovery once these collections have been thoroughly characterized genotypically and phenotypically for diverse traits of agronomic importance, including yield, (a)biotic stress tolerance, and nutritional quality traits. Under this perspective, candidate gene-based association mapping and genome-wide association studies (GWAS) relying on the large-scale genotyping of informative SNP markers and robust field phenotyping information on these core or mini-core germplasm lines (i.e., association panels) are now considered to be an effective approach for the identification of major and minor genes/QTLs and alleles regulating both simple qualitative and complex quantitative traits in grain legume crop plants (Varshney et al., 2011). Candidate gene-based association mapping, which is carried out using genotyping information from SNPs in various coding and regulatory sequence components of genes in a trait-specific association panel, plays a significant role in the identification of genes/QTLs controlling yield, nutritional quality, and stress tolerance traits in grain legume crops. With the availability of high-throughput genome-wide SSR and SNP marker-based genotyping information on germplasm lines belonging to an association panel, the GWAS has now become a routine approach for high-resolution scanning of the whole genome to identify target genomic regions, including genes/QTLs (both major and minor) associated with traits of agricultural importance in many grain legume crops (Varshney et al., 2017; Varshney et al., 2021). The trait-influencing molecular signatures once identified using trait association mapping are significant for their potential utilization for genomics- (marker-)assisted crop improvement programs.

The delineated molecular signatures regulating traits of agronomic importance have been utilized for introgression, combining, and pyramiding into selected grain legume crop genotypes of interest through traditional and advanced genomics-assisted breeding approaches in order to develop superior crop varieties in terms of yield and stress tolerance (Varshney, 2016). Recently, a chickpea cultivar ‘Pusa JG16’ has been released in India as a drought-hardy cultivar; this was developed through genomics-assisted breeding utilizing a QTL-hotspot region from ICC4958 (Bhardwaj et al., 2021). Introgressions of functional natural genetic variations and of favorable genes, QTLs, alleles, and chromosomal segments identified from a larger set of grain legume germplasm accessions (including landraces and wild species), particularly for yield and stress component traits, have been transferred into the cultivated genetic background for improvement of the relevant crop through the use of such approaches as introgression lines (ILs), advanced-backcross QTL (AB–QTL) analysis, association genetics, and multi-parent advanced generation intercross (MAGIC) populations (Roorkiwal et al., 2020; Bohra et al., 2021). For example, the ‘Geletu’ chickpea variety was developed through marker-assisted back-crossing (MABC) and released in Ethiopia; it provided a yield advantage of 15% over the check variety ‘Teketay’ and 78% over a local check (https://www.icrisat.org/). The MABC technique has been used in the development of introgression of QTLs into elite cultivars in order to develop introgressed lines (Bharadwaj and Yadav, 2012; Varshney et al., 2013b; Barmukh et al., 2022). The ‘Pusa Chickpea 20211’ variety is another example in which resistance genes for multiple races (*foc* 1,2,3,4, and 5) of fusarium wilt have been stacked through MABC in the mega desi chickpea variety ‘Pusa 391’ (Bharadwaj et al., 2022). Molecular tags associated with major effects on qualitative and quantitative trait regulation have now been transferred into diverse grain legume crop genotypes for their genetic enhancement through marker-assisted selection (MAS), including MABC and marker-assisted foreground and background selection. The identification of a QTL-hotspot region in linkage group 4 (CaLG04) in chickpea that harbors major QTLs for multiple drought adaptive traits, followed by its introgression into elite chickpea cultivars, is an excellent example of genomics-assisted breeding (Barmukh et al., 2022). This region accounts for 58.2% of explained phenotypic variation and a 16% yield enhancement under drought conditions in introgressed lines, which is primarily attributed to improvements in root traits, such root length, density, surface area, and volume (Varshney et al., 2013b; Roorkiwal et al., 2020; Bharadwaj et al*.*, 2021).

Complications in the domain of genetic background effects/epistasis and linkage drag of QTLs, as well as minor effects of both minor and major QTLs/genes on complex trait regulation, have impeded the use of the traditional MAS (QTL–MAS) approach in the genetic enhancement of grain legume crops on complex quantitative traits. To overcome these complexities, many novel advanced genomics-assisted breeding approaches are currently emerging, such as marker-assisted recurrent selection (MARS), MAGIC, and genomic/genome-wide (haplotype) selection. These involve the transferal and pyramiding of the favorable alleles of minor-effect genes/QTLs controlling complex quantitative traits for the genetic enhancement of grain legume crop plants in terms of yield, nutritional quality, and stress tolerance. Varshney et al. (2021) have identified superior haplotypes through whole genome sequencing (WGS) of 3,336 accessions, both cultivated (3,171) and wild (195), for important traits relating to yield enhancement; these can then be introgressed into elite chickpea cultivars. The study also identified target genomic regions for the purging of deleterious alleles, which can be achieved through genomics-assisted breeding and/or gene editing (Varshney et al., 2021). Similarly, superior haplotypes have been identified in pigeon pea based on the 292 pigeon pea genotypes of a reference set that included breeding lines, landraces, and wild species (Sinha et al., 2020). In this study, haplotype–phenotype association analysis for drought-responsive traits resulted in the identification of promising haplotypes (*C. cajan_23080*-H2, *C. cajan_30211-*H6, *C. cajan_26230*-H11, and *C. cajan_26230*-H5) for three genes regulating five drought component traits (Sinha et al., 2020). Genomic selection (GS) and integrated genomic–enviromic prediction (iGEP) are other promising strategies that can be used to improve genetic gain in legume crops (Heffner et al., 2009; Xu et al., 2022). GS uses a smaller training population that is well genotyped and phenotyped, while iGEP uses additional data on genotype–environment interactions to build a prediction model. These models are then used to predict the true breeding values of selecting particular candidates based on multi-omics data, big data technology, and artificial intelligence (Xu et al., 2022). Thus, genomics plays an integral role in improving genetic gain in modern agricultural practices and has immense potential to expedite future grain legume crop improvement programs. The traditional and novel genomics-assisted breeding approaches that are now available provide clues to the quantitative dissection of complex trait regulation, and thus have potential to expedite studies of the genetic enhancement of complex traits in diverse grain legume crops.

## 5 Future outlook

Grain legumes are a major source of food and nutrition globally. However, it has not been possible to enhance gain legume production to the required level, primarily due to the narrow genetic base of most of the legume crops, coupled with changing climatic conditions. Most grain legumes are lacking in desired plant ideotypes and resistance sources for various biotic and abiotic stresses. The genetic diversity conserved in gene banks globally is a major resource for crop breeding programs, but it is utilized only marginally. Therefore, in order to broaden the genetic base of grain legume crops and enhance the genetic gains made in improvement programs, conventional approaches and modern scientific tools should be integrated in a phased and carefully judged manner. The first phase should focus on the search for desired traits and the infusion of diversity into the cultivated gene pool through use of landraces and CWRs. The second phase should focus on the utilization of advanced selection tools, such as genomics, high-throughput precision phenotyping, and artificial intelligence, to exploit the hidden potential of the available genetic diversity; and the third phase should involve technologies such as mutational breeding, genome editing, and transgenic technologies to improve, modify, or introgress any novel or alien traits that are not available in the entire crop gene pool. We presume that enrichment of the genetic diversity of cultivated grain legume gene pools, along with simultaneous improvements in their yield and plant type with the aid of advanced scientific tools, will enhance grain legume crop yields to the required level.
